# New insights into the existing image encryption algorithms based on DNA coding

**DOI:** 10.1371/journal.pone.0241184

**Published:** 2020-10-23

**Authors:** Xianglian Xue, Dongsheng Zhou, Changjun Zhou

**Affiliations:** 1 Section of Computer Teaching and Research, Shaanxi University of Chinese Medicine, Xianyang, China; 2 Laboratory of Network Computer and Security Technology in Shaanxi Province, Xi’an University of Technology, Xi’an, China; 3 Key Laboratory of Advanced Design and Intelligent Computing, Ministry of Education, Dalian University, Dalian, China; 4 College of Mathematics and Computer Science, Zhejiang Normal University, Jinhua, China; Lanzhou University of Technology, CHINA

## Abstract

Because a DNA nucleotide sequence has the characteristics of large storage capacity, high parallelism, and low energy consumption, DNA cryptography is favored by information security researchers. Image encryption algorithms based on DNA coding have become a research hotspot in the field of image encryption and security. In this study, based on a comprehensive review of the existing studies and their results, we present new insights into the existing image encryption algorithms based on DNA coding. First, the existing algorithms were summarized and classified into five types, depending on the type of DNA coding: DNA fixed coding, DNA dynamic coding, different types of base complement operation, different DNA sequence algebraic operations, and combinations of multiple DNA operations. Second, we analyzed and studied each classification algorithm using simulation and obtained their advantages and disadvantages. Third, the DNA coding mechanisms, DNA algebraic operations, and DNA algebraic combination operations were compared and discussed. Then, a new scheme was proposed by combining the optimal coding mechanism with the optimal DNA coding operation. Finally, we revealed the shortcomings of the existing studies and the future direction for improving image encryption methods based on DNA coding.

## 1 Introduction

Since Dr. Adleman of the United States used DNA molecular biological computing to solve the directed path problem of seven nodes [1] in 1994, DNA computing has attracted the attention of researchers across the world [2–5]. In DNA computing, DNA nucleotide bases A, C, G, and T coding sequences are used as carriers of information; it has great advantages in dealing with large storage capacity, parallelism, and energy consumption of information [6–9]. DNA computing essentially uses biochemical experiments to address practical problems. However, because of the limitations of biochemical reaction conditions, such as expensive experimental equipment, environmental requirements, difficulty in extracting DNA sequence, and difficulties in controlling the concentration, temperature, and PH of the reactant, studying DNA computing is difficult. Regarding image encryption in DNA computing, researchers ignore the complex experimental links of DNA, only use DNA coding to carry image information, and design a reasonable and effective encryption algorithm by combining chaotic mapping with different DNA coding algorithms. This idea gives rise to new perspectives in the research on image encryption.

From 2009 to 2014, Zhang et al. innovatively used DNA coding methods for image encryption, and proposed many image encryption algorithms based on DNA coding [10–17]; this created new ideas in DNA cryptography. Their main encryption ideas involve the following four steps. First, the original image is encoded using the DNA encoding rule. Then, the position of the DNA-encoded matrix is scrambled by the chaotic sequences generated by the one-dimensional chaotic system [14], combination of several low-dimensional chaotic systems [10–12], and hyper chaotic system [13, 15] or the combination of several hyper chaotic systems [16, 17]. Subsequently, the values of the image pixels are changed by various operations, namely, DNA addition and subtraction [10, 12, 13], XOR [15], DNA subsequence [16], DNA complement [11], or the combination of these operations [14, 17] under the control of chaotic sequences. Finally, the encrypted image is obtained by DNA decoding and regrouping. The image encryption technology based on DNA coding proposed by Zhang et al. combined DNA coding and image encryption effectively. Consequently, the application of DNA cryptography in image encryption garnered the attention of many researchers both home and abroad. However, in recent years, researchers have highlighted many shortcomings in the methods proposed by Zhang et al. For example, [18, 19] observed that the method of Zhang [12] combined DNA addition with chaotic mapping to encrypt an image. Nevertheless, the addition operation of this method is non-invertible; therefore, the decrypted image cannot be obtained. Belazi [20], Liu [21], and Wang [22] used an RGB image encryption algorithm based on DNA coding and chaotic mapping proposed by Liu and Zhang [14], to restore original images using chosen-plaintext attack (CPA). Wang indicated that the main drawback of this algorithm is that the key used for the encryption is independent of the original image. Furthermore, changing a base in DNA can only affect the base in one position; moreover, the diffusion ability of the base is poor, resulting in poor diffusion ability of the image pixels. Similarly, an image fusion encryption algorithm based on the DNA sequence operation and hyper chaotic system proposed by Zhang and Guo et al. [15] was broken by choosing different sizes of plain images in [23–26]. Li [27] analyzed some image encryption algorithms based on the DNA subsequence operation and chaotic system proposed by Zhang [16] using CPA to obtain original images. In summary, although the methods proposed by Zhang et al. are effective, their security is very poor.

Since 2015, some researchers have applied a combination of multiple simple chaos, more complex, or more secure chaotic systems to encrypt images, for enhancing the security. For instance, Wu [28] used three one-dimensional chaotic systems combined with the DNA addition and subtraction, and XOR operations. Zhang [29] and Mondal [30] used a mixed linear–nonlinear coupled map lattices (MLNCML) system embedded logistic, and Zhang [31] used a fractional-order piecewise-linear (PWL) hyperchaotic map combined with DNA addition and subtraction, to realize image encryption. Li [32] used fractional-order hyper chaotic mapping Lorenz (FOHCL) to determine the rules of DNA addition and subtraction as well as DNA XOR. Color images are encrypted using a logistic system in combination with DNA addition and subtraction, and XOR [33]. Similarly, Zhang [34] used a logistic system combined with DNA addition, subtraction, and complementation. The DNA coding method used in the above-mentioned algorithms is the same as that in the method of Zhang et al., which adopts fixed DNA coding, and gives DNA coding operation rules (DNA addition and subtraction, DNA XOR, and DNA complementation). The security of the above-mentioned encryption algorithms is guaranteed by a chaotic system. Without a chaotic system, the image encryption based on DNA would only be a binary bit computation. If the security is greatly threatened, it is difficult to resist the brute-force attack, known plaintext attack (KPA), and CPA. For instance, Dou [35] used brute-force attack and CPA to analyze Ref. [30], and restored the plaintext image. In addition, Kumar [36] used DNA encoding and elliptic curve Diffie–Hellman cryptography without a chaotic system to realize RGB image encryption. However, this method was less-secure, and was broken by Akhavan [37] via CPA.

For these reasons, dynamic DNA coding and more complex DNA coding algorithms have been proposed in recent years. Kalpana [38], Zhang [39], Zhen [40], Cai [41], Rehman [42], and many others proposed various dynamic DNA coding algorithms. They first defined different DNA coding rules, and used chaotic sequences to select DNA coding rules dynamically to encode images. Further, [39, 43, 44] proposed replacing the conventional single-base complement operation with a complement operation based on the principle of base complementation, to increase the complexity of DNA operation. These encryption algorithms achieved better encryption results. Belazi [45] proposed a novel medical image encryption scheme based on chaos and DNA encoding, and realized two encryption rounds using different coding rules and complement, and XOR operation under chaotic control. This method achieves good encryption, and could resist all types of attacks. Furthermore, a new image encryption scheme based on CML and DNA sequences was proposed by Wang [46], which also performs good encryption. Nevertheless, because fixed DNA coding was selected in their coding process, and the security could be improved. Dagadu [47] proposed a medical image encryption scheme based on multiple chaos and DNA coding, different DNA coding rules, and XOR operation combined with chaotic map to realize image encryption. Although the scrambling degree in their method was high, it could not resist CPA and KPA.

This literature review reveals that researchers generally improve the performance of the encryption algorithms by changing the DNA coding methods and operations. The existing studies simply use a DNA coding method, DNA coding operation (addition, subtraction, complement, XOR, etc.), or a combination of multiple coding operations to realize image encryption, without comprehensively comparing or analyzing the methods or operations. Put differently, in any existing study, the reasons for selecting a specific DNA coding (fixed or dynamic coding) method, DNA coding operation, or a combination of multiple coding operations to realize image encryption are unclear.

Therefore, this study provides new insights into the existing image encryption algorithms based on DNA coding. We categorized the existing image encryption algorithms based on DNA coding into five types, depending on the type of DNA coding: fixed DNA coding, dynamic DNA coding, different types of DNA base complement operations, different DNA sequence algebraic operations, and combinations of multiple DNA operations. Then, a more detailed classification is performed according to the characteristics of the different algorithms. Furthermore, we comprehensively compared and analyzed all these methods, and revealed their advantages and disadvantages. Further, the DNA coding mechanism, DNA algebraic operation, and DNA algebraic combination operation are compared, and the newly proposed scheme is discussed. Finally, the study highlights the shortcomings and indicates the future research direction for improving image encryption based on DNA coding.

The remainder of this manuscript is structured as follows. In the second section, the theoretical basis of this study is introduced. In the third section, we categorize the existing DNA coding-based image encryption methods, and discuss their advantages and disadvantages by a comprehensive comparison and analysis, and on the basis of above discussion a new method is proposed. Finally, the last section discusses the shortcomings and the future research direction for improving image encryption based on DNA coding.

## 2 Theoretical basis of this study

### 2.1 DNA coding

A DNA sequence is composed of four nucleic acid bases, namely, adenine (A), cytosine(C), guanine (G), and thymine (T), wherein A and T, and G and C are complements. Researchers use the binary values 00, 01, 10, and 11 to denote these four bases. A total of 24 types of coding can be listed; however, according to the complementary relation between 0 and 1 in binary, it can be deduced that 00 and 11 are complements, and that 01 and 10 complement each other. Therefore, 8 out of the 24 coding rules are selected to satisfy the base complementary criterion shown in Table 1. The pixel value of an image lies between [0, 255], and consists of eight-bit binary numbers; therefore, a single pixel value may consist of four-bit DNA bases. For example, for a pixel value of 175, the corresponding binary bit is “10101111,” the DNA sequence generated by the R1 rule presented in Table 1 is “GGTT,” and decoding is performed with the same rule or with the other seven different rules. The existing image encryption algorithms based on DNA coding are almost inseparable from this coding rule or deforming based on this coding rule.

**Table 1:** Eight types of DNA coding rules.

Binary	R1	R2	R3	R4	R5	R6	R7	R8
00	A	A	C	C	G	G	T	T
01	C	G	A	T	A	T	C	G
10	G	C	T	A	T	A	G	C
11	T	T	G	G	C	C	A	A

### 2.2 DNA complement rules

Two methods are available for image encryption based on DNA sequence complement: (1) single base direct complement method, and (2) the method that uses the principle of single base and double base complementary pairing in biotechnology to perform the complement operation. A single base direct complement is defined as follows:

$$\begin{array}{c} \left\{ \begin{array}{c} T=complement(A)\\ A=complement(T)\\ G=complement(C)\\ C=complement(G) \end{array}\right. \end{array}$$ (1)

where complement (.) is the function of the complement. The complement of base A is T, and that of base C is G. The corresponding binary complement is satisfied when both the complement of 00 is 11 and that of 01 is 10, and vice versa.

The complement rule is defined based on a double helix structure; a nucleoside is paired according to the double helix structure. Supposing the complementary transformation is D, every nucleoside *x*_*i*_ satisfies the following equation:

$$\begin{array}{c} \left\{ \begin{array}{c} x_{i}\neq D\left(x_{i}\right)\neq D\left(D\left(x_{i}\right)\right)\neq D\left(D\left(D\left(x_{i}\right)\right)\right)\\ x_{i}=D\left(D\left(D\left(D\left(x_{i}\right)\right)\right)\right) \end{array}\right. \end{array}$$ (2)

where *x*_*i*_ and *D*(*x*_*i*_) are complementary, i.e., *x*_*i*_ and *D*(*x*_*i*_) are a pair of base pairs. These base pairs must satisfy the condition of single-shot mapping. Based on the above equation, the base pairs satisfying single-shot mappings are listed in Table 2 [48].

**Table 2:** Complement operation for base complementary principle.

Rules	Complement operation			
R1	(AT)	(TC)	(CG)	(GA)
R2	(AT)	(TG)	(GC)	(CA)
R3	(AC)	(CG)	(GT)	(TA)
R4	(AC)	(CT)	(TG)	(GA)
R5	(AG)	(GC)	(CT)	(TA)
R6	(AG)	(GT)	(TC)	(CA)

### 2.3 DNA addition, subtraction, and XOR algebra operations

Because encoding and decoding processes are implemented in binary, the algebraic operation of the DNA sequence (addition or subtraction) follows the binary operation rule. The coding of eight different rules corresponds to eight different operations (addition or subtraction). In the processes of encryption and decryption, the addition and subtraction operations are reciprocal. Furthermore, the inverse operation of XOR remains an XOR operation. Therefore, we only list the addition and XOR operations, and Table 3 is based on the addition and XOR operations of R1 in Table 1.

**Table 3:** DNA sequence of addition and XOR operations.

+	A	C	G	T	XOR	A	C	G	T
A	A	C	G	T	A	A	C	G	T
C	C	G	T	A	C	C	A	T	G
G	G	T	A	C	G	G	T	A	C
T	T	A	C	G	T	T	G	C	A

### 2.4 Correlation equation of this study

For a comprehensive comparison, we use the logistic chaotic sequence under the same parameter to act on the operation of the DNA sequence. Additionally, histogram, information entropy [49], rate of base distribution, hamming distance [50], fixed point ratio [51], histogram variance [52], correlation coefficient, and the number of pixel change rate (NPCR) and unified average changing intensity (UACI) are used as the evaluation indexes. The relevant theories of these evaluation indexes are given by the following Eqs (3)–(16).

1. Logistic map

A logistic map is an excellent chaotic map. A logistic map is used in this study for experimental simulation, and is described as follows:

$$\begin{array}{c} x_{n+1}=\mu x_{n}\left(1-x_{n}\right) \end{array}$$ (3)

where *μ* ∈ [0, 4], *x*_*n*_ ∈ (0, 1), *n* = 0, 1, 2…,. The study result showed that the system is in a chaotic state under the condition 3.569945 < *μ* ≤ 4.

2. Global information entropy and local information entropy

The global information entropy is defined below.

$$\begin{array}{c} H\left(m\right)=-\sum_{i=0}^{L}P\left(m_{i}\right)log_{2}P\left(m_{i}\right) \end{array}$$ (4)

where *m*_*i*_ denotes the *ith* grey value for the *L* level grey image, and *P*(*m*_*i*_) represents the emergence probability of *m*_*i*_. The information entropy of an ideal random image is eight.

The local information entropy is defined below.

In a test image S, we select the non-overlapping image blocks *S*_1_, *S*_2_, *S*_3_…*S*_*k*_ randomly, and calculate the information entropy of each image block using Eq (4). Here, the intensity scale of the test image is L; generally, L = 256. Finally, the following equation is used to calculate the average value of the information entropy of the image blocks.

$$\begin{array}{c} H{\left(S\right)}_{local}=\sum_{i=1}^{k}\frac{H(S_{i})}{k} \end{array}$$ (5)

where *H*(*S*)_*local*_ denotes the local information entropy of the image blocks, and i = 1,2,3…k.

3. Rate of base distribution

The rate of base distribution is defined below.

$$\begin{array}{c} AP=count(A)÷(M\times N\times 4)\times 100\% \end{array}$$ (6)

where count (A) denotes the number of bases “A” in the entire image coding matrix; M ×N denotes the size of the image (a pixel can be represented by four bases; therefore, M×N×4 is the total number of bases); and AP denotes the percentage of base “A” in the entire coding matrix (the distribution of other bases is similar). Because DNA coding consists of four different bases (A, C, G, and T), the distribution rate of the DNA bases in an ideal random image should be 25%.

4. Hamming distance

The hamming distance is used to calculate the total number of different bases at the same location for two sequences of equal length, and is given by,

$$\begin{array}{c} \left\{ \begin{array}{c} D\left(M,N\right)=\sum_{i=0}^{L}d\left(m_{i},n_{i}\right)\\ d\left(m_{i},n_{i}\right)=\{\begin{array}{cc} 0, & if\;m_{i}=n_{i}\\ 1, & if\;m_{i}\neq n_{i} \end{array} \end{array}\right. \end{array}$$ (7)

where *m*_*i*_ and *n*_*i*_ denote the *ith* base of the DNA sequence M and N, respectively, and D(M,N) denotes the hamming distance of M and N; the greater the hamming distance, the greater the difference between the bases in the two sequences.

5. Fixed point ratio

Let *O* = (*O*_*i*,*j*_)_*M*×*N*_ and *N* = (*N*_*i*,*j*_)_*M*×*N*_ represent the original and encrypted images, respectively, where *M* × *N* defines their sizes. If the pixel position (i,j) in an image O does not change its gray value after scrambling (i.e., *o*_*ij*_ = *n*_*ij*_), the pixel is a fixed point. We use the following equation to represent the fixed point ratio:

$$\begin{array}{c} FP\left(O,N\right)=\frac{\sum_{i=1}^{M}\sum_{j=1}^{N}f\left(i,j\right)}{M\times N}\times 100\% \end{array}$$ (8)

If *o*_*ij*_ = *n*_*ij*_, *f*(*i*, *j*) = 1, and if *o*_*ij*_ ≠ *n*_*ij*_, *f*(*i*, *j*) = 0; clearly, the smaller the fixed point ratio of the two images, the greater the difference between the encrypted image and the original image and the better the scrambling effect.

6. Histogram variance

In addition to visual analysis to determine the distribution of image histograms, the histogram variance can be used for quantitative analysis. It is known that visual analysis is often unreliable. The histogram variance is defined below.

$$\begin{array}{c} Var\left(M\right)=\frac{1}{n^{2}}\times \sum_{i=1}^{n}\sum_{j=1}^{n}\frac{1}{2}{\left(m_{i}-m_{j}\right)}^{2} \end{array}$$ (9)

where *M* = *m*_0_, *m*_1_, …, *m*_255_ denotes the vector of the histogram value, *m*_*i*_ and *m*_*j*_ denote the numbers of pixels whose gray values are given by i and j, respectively, and n is the greyness level. Clearly, the smaller the histogram variance, the more uniform the histogram distribution of the image.

7. Correlation coefficient

The correlation of the adjacent pixels in an original image is very high. Therefore, the better the encryption, the smaller the correlation coefficient (close to 0). In this study, we randomly select 8000 pairs (horizontal, vertical, and diagonal) of adjacent pixels from the original and encrypted images. Then, we use the following equations [53] to calculate the correlation coefficient:

$$\begin{array}{c} E\left(x\right)=\frac{1}{N}\times \sum_{i=1}^{N}x_{i} \end{array}$$ (10)

$$\begin{array}{c} D\left(x\right)=\frac{1}{N}\times \sum_{i=1}^{N}\left(x_{i}-E{\left(x\right)}^{2}\right) \end{array}$$ (11)

$$\begin{array}{c} cov\left(x\right)=\frac{1}{N}\times \sum_{i=1}^{N}\left(x_{i}-E\left(x\right)\right)\left(y_{i}-E\left(y\right)\right) \end{array}$$ (12)

$$\begin{array}{c} r_{xy}=\frac{cov(x,y)}{\sqrt{D(x)}\sqrt{D(y)}} \end{array}$$ (13)

where *x* and *y* denote the grey value of two adjacent pixels in the image.

8. Analysis of differences between two images

The NPCR and UACI are used in this study to analyze the differences between two images. They are defined as follows [54]:

$$\begin{array}{c} NPCR=\frac{\sum_{i=0}^{M-1}\sum_{j=0}^{N-1}D\left(i,j\right)}{M\times N}\times 100\% \end{array}$$ (14)

$$\begin{array}{c} D\left(i,j\right)=\{\begin{array}{cc} 0, & if\;E\left(i,j\right)=E^{{}^{\prime }}\left(i,j\right)\\ 1, & if\;E\left(i,j\right)\neq E^{{}^{\prime }}\left(i,j\right) \end{array} \end{array}$$ (15)

$$\begin{array}{c} UACI=\frac{\sum_{i=0}^{M-1}\sum_{j=0}^{N-1}\frac{|E\left(i,j\right)-E^{\prime }\left(i,j\right)|}{255}}{M\times N}\times 100\% \end{array}$$ (16)

## 3 Analysis and comparison of the existing DNA coding-based image encryption methods

**Fig 1:**
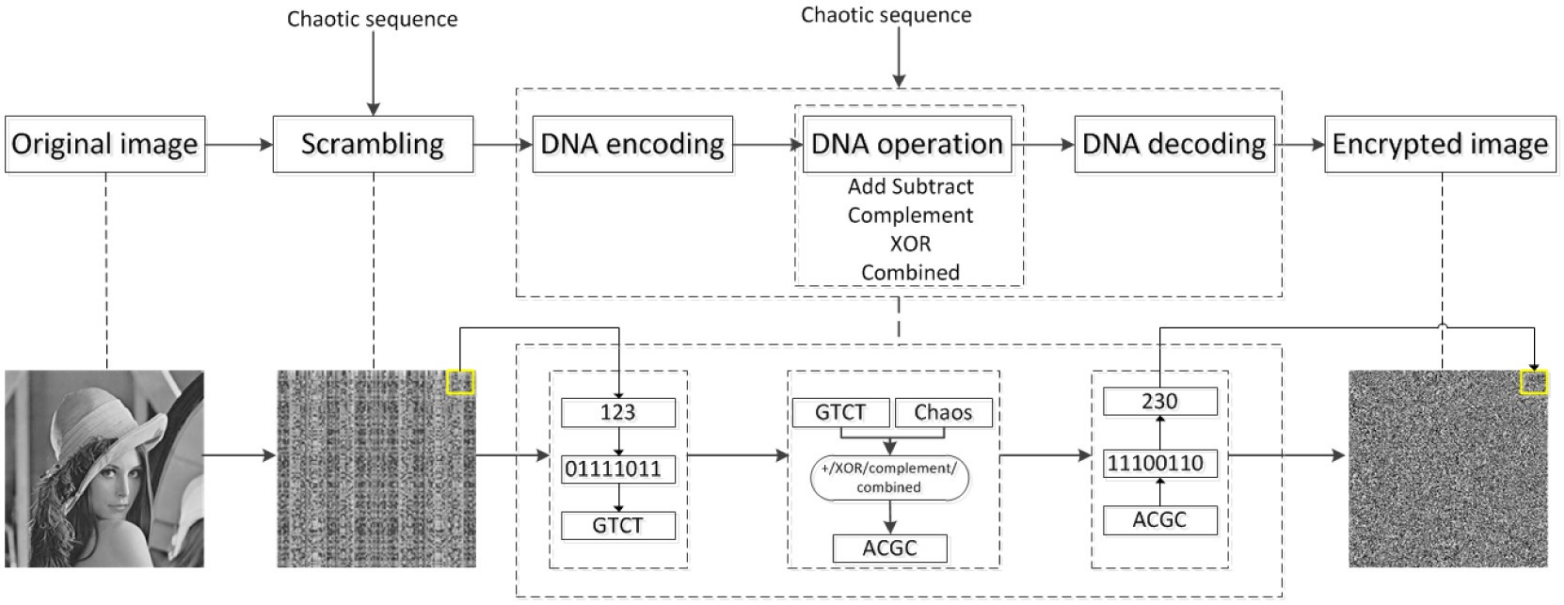
Block diagram of image encryption based on DNA coding.

The existing image encryption algorithms based on DNA coding involve four basic processes: (1) scrambling the pixel position of the image by using a chaotic sequence; (2) encoding the scrambled image matrix to the DNA sequence; (3) disturbing the DNA sequence matrix by using a chaotic sequence combined with addition, subtraction, XOR, or complement operation, or a combination of these operations; (4) obtaining the encrypted image by DNA decoding and recombination. The block diagram of these processes is shown in Fig 1. As discussed already, a DNA chain consists of four bases (A, C, G, and T), which are used as the carriers of information. The process of converting the information into a DNA nucleotide chain is known as encoding. The opposite process of converting a DNA nucleotide into information is known as decoding. These processes are depicted in Fig 1. DNA encoding and decoding are the key problems in image encryption based on DNA coding. As discussed already, a DNA operation includes addition, subtraction, XOR, and complement. Apparently, different types of encoding, operations, and decoding can produce different encryption results. Therefore, researchers use different methods of DNA encoding and decoding, and different DNA operations, to achieve safer and more effective image encryption.

**Fig 2:**
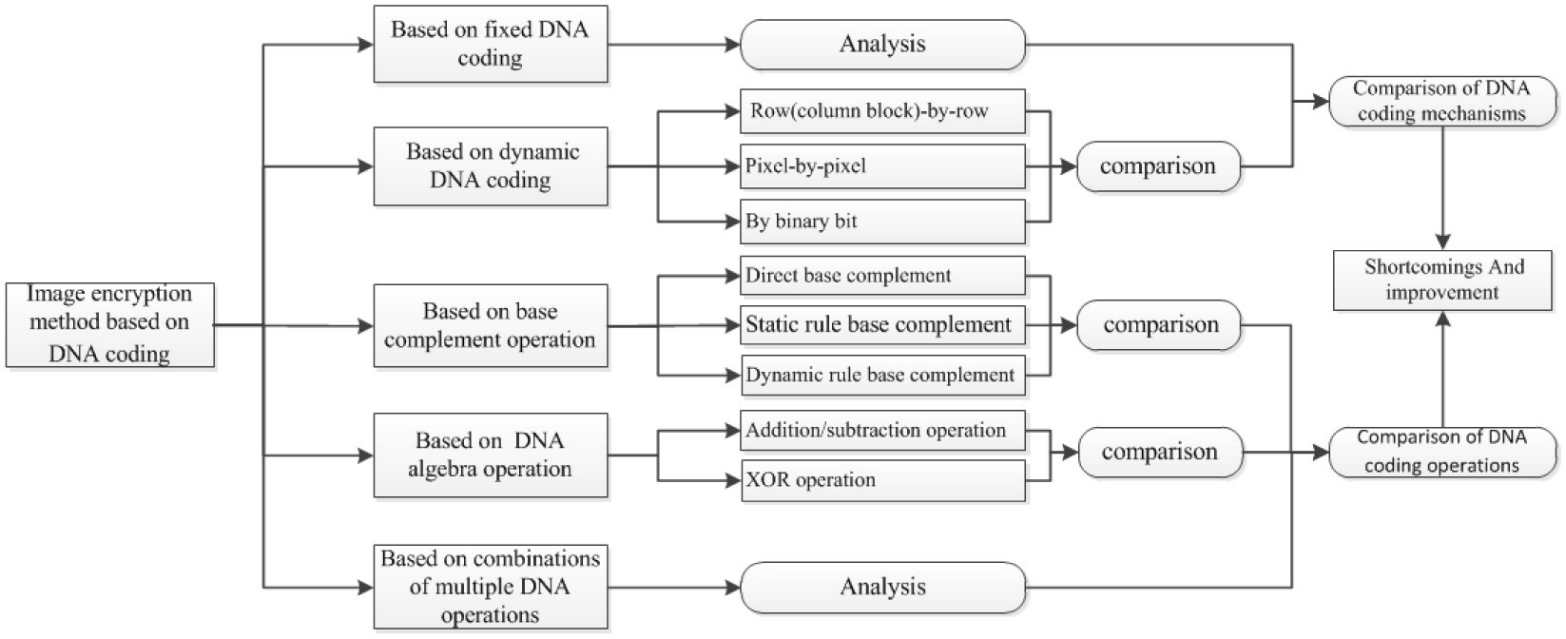
The framework of this research.

Now, the existing image encryption algorithms based on DNA coding are categorized into five, depending on the type of DNA coding: fixed DNA coding, dynamic DNA coding, different types of base complement operations, different DNA sequence algebra operations, and combinations of multiple DNA operations. Dynamic DNA coding is further classified into three categories: image block or row-column dynamic coding (row-by-row), pixel dynamic coding (pixel-by-pixel), and binary bit dynamic coding. The image encryption based on different types of base complement operations is categorized into two: single base direct complement method and base complementary method based on the principle of base complement (static regular base complement and dynamic regular base complement). Further, addition and subtraction operations and the XOR operation based on different DNA sequence algebra operations are available. We not only analyzed and compared all the five methods independently, but also compared and studied the DNA coding mechanism, DNA coding operation, and DNA combination operation intensively. The specific classification scheme is shown in Fig 2.

### 3.1 Image encryption based on fixed DNA coding

Researchers typically use a DNA coding rule listed in Table 1 for encoding; after performing some DNA operations, the DNA sequence is decoded using the same encoding rule or other encoding rules listed in Table 1. We refer to the way of coding as the fixed DNA coding method in this study. The previous studies [55–58] adopted a specific coding rule for encoding, and used the same rule for decoding. For example, for the pixel value of 175, the corresponding binary bit is “10101111,” the DNA sequence generated using the R1 rule defined in Table 1 is “GGTT,” and decoding is performed using the same rule. Apparently, multi-step encryption operations must be performed before decoding. However, [10–14, 16, 17] randomly selected one of the eight coding rules for encoding, and selected other rules for decoding using seed key1 and key2. For instance, the pixel value of 175 was encoded with the R1 rule to generate the DNA sequence “GGTT,” and “GGTT” was decoded using the R5 rule to obtain the binary bit “00001010”; the corresponding decimal number is 10. Apparently, the pixel values are changed in this manner.

To further analyze and study the image encryption algorithm based on DNA fixed coding, we conducted two experiments.

**Fig 3:**
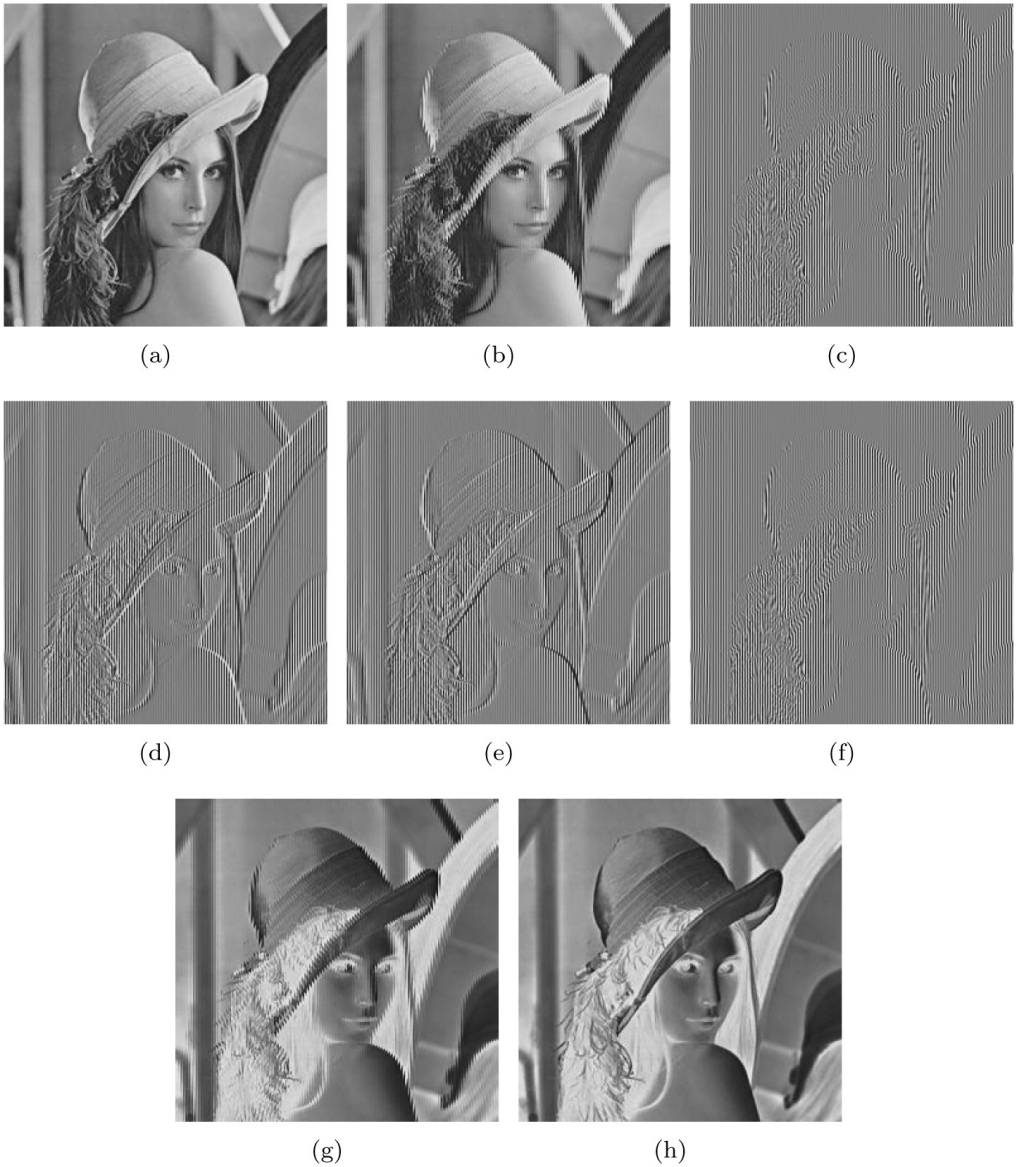
Encoding of original image using the R1 rule and decoding it using the remaining seven rules to obtain the encrypted image: (a)Original image. (b) Encoding with R1 and decoding with R2. (c) Encoding with R1 and decoding with R3. (d) Encoding with R1 and decoding with R4. (e) Encoding with R1 and decoding with R5. (f) Encoding with R1 and decoding with R6. (g) Encoding with R1 and decoding with R7. (h) Encoding with R1 and decoding with R8.

Experiment 1: A “lena” image whose size is 256×256 was first encoded using the R1 rule defined in Table 1. Then, the encrypted image was obtained by decoding using the other seven rules. The results are shown in Fig 3.

Experiment 2: Eqs (4), (6) and (8) are used to calculate the base distribution, information entropy, and histogram variance of the eight different coding. Then, the time complexity of the algorithm and the maximum distance between the base distribution and 25% are calculated. The results are presented in Table 4.

**Table 4:** Comparison of the characteristics of the eight different fixed coding.

Comparative characteristic	R1	R2	R3	R4	R5	R6	R7	R8
AP	35.60%	35.60%	15.24%	15.31%	15.24%	15.31%	33.85%	33.85%
TP	33.85%	33.85%	15.31%	15.24%	15.31%	15.24%	35.60%	35.60%
CP	15.24%	15.31%	35.60%	35.60%	33.85%	33.85%	15.24%	15.31%
GP	15.31%	15.24%	33.85%	33.85%	35.60%	33.60%	15.31%	15.24%
Information entropy	7.4603	7.4603	7.4894	7.4894	7.4894	7.4894	7.4603	7.4603
Histogram variance	40068	40068	36189	36189	36189	36189	40068	40068
Complexity order	*O*(8*MN*)	*O*(8*MN*)	*O*(8*MN*)	*O*(8*MN*)	*O*(8*MN*)	*O*(8*MN*)	*O*(8*MN*)	*O*(8*MN*)
The max distance of base distribution and 25%				10.6%				

From the analysis of the test results of Experiment 1, the results of encoding by R1 and decoding by the other seven rules are shown in Fig 3. In fact, Fig 3(a) can be regarded as an image encoded by R1 and decoded by R1. The results shown in Fig 3(a), 3(b), 3(d), 3(e), 3(c), 3(f), 3(g) and 3(h) are similar based on visual observations. Therefore, it can be concluded that among the eight types of coding mechanisms, only four are effective. The contour of the original image can be observed in all the encrypted images. Table 4, which presents the results of Experiment 2, shows that the base distributions of the eight coding rules are not uniform. The maximum distance between the base distribution and 25% is 10.6%, and the base distributions of R1 and R2, R3 and R4, R5 and R6, and R7 and R8 are very similar. Their information entropy is not close to eight, and the histogram variance is very large. However, the complexity order is only *O*(8*MN*) (*M*, *N* denotes the size of the image).

Therefore, the following conclusions can be drawn: fixed DNA coding rules are simple to implement, have high computational efficiency, and one can even disturb a pixel value by selecting decoding rules that are different from the encoding rules. However, because there are only eight coding combinations, effective results are obtained only for four kinds. Further, the encryption is poor, ability to resist exhaustion is poor, bit distribution of the bases is not uniform, and the degree of scrambling is low. Therefore, it is difficult to encrypt an image, especially a single-pixel image such as a medical image. To our knowledge, most existing image encryption algorithms based on DNA coding adopt fixed coding; therefore, their security is apparently under threat.

### 3.2 Image encryption based on dynamic DNA coding

In dynamic DNA coding of images, the DNA sequence is obtained using different rules (the eight rules listed in Table 1) to encode each row, each column, each pixel, or each binary bit of the whole image. Selecting encoding patterns for different encoding objects randomly makes the coding system more complex, renders decoding more difficult, and enhances the image encryption security.

In this study, the existing dynamic coding methods are categorized into three, as follows. (1) Dynamic coding according to image block or row–column (row(column/block)-by-row). For example, Zhen [40] encoded each row of the original image differently using different rules that were controlled by a logistic chaotic sequence, and obtained the encrypted image by decoding with one of the rules. (2) Dynamic coding according to pixel (pixel-by-pixel). For instance, Kalpana [38], Wang [48], and Dagadu et al. [47] chose different encoding rules to encode each pixel in the image under the action of chaotic sequences. (3) Dynamic coding according to binary bit (bit-by-bit), wherein a single pixel can be converted to 8-bit binary bits, and every two binary bits can be encoded into one base using different rules that are controlled by the chaotic sequence; this method was used by [39]. The decoding process of all the above three methods is simply the opposite of the encoding process, and is thus not discussed here.

Now, the three dynamic DNA coding methods are comprehensively analyzed.

**Fig 4:**
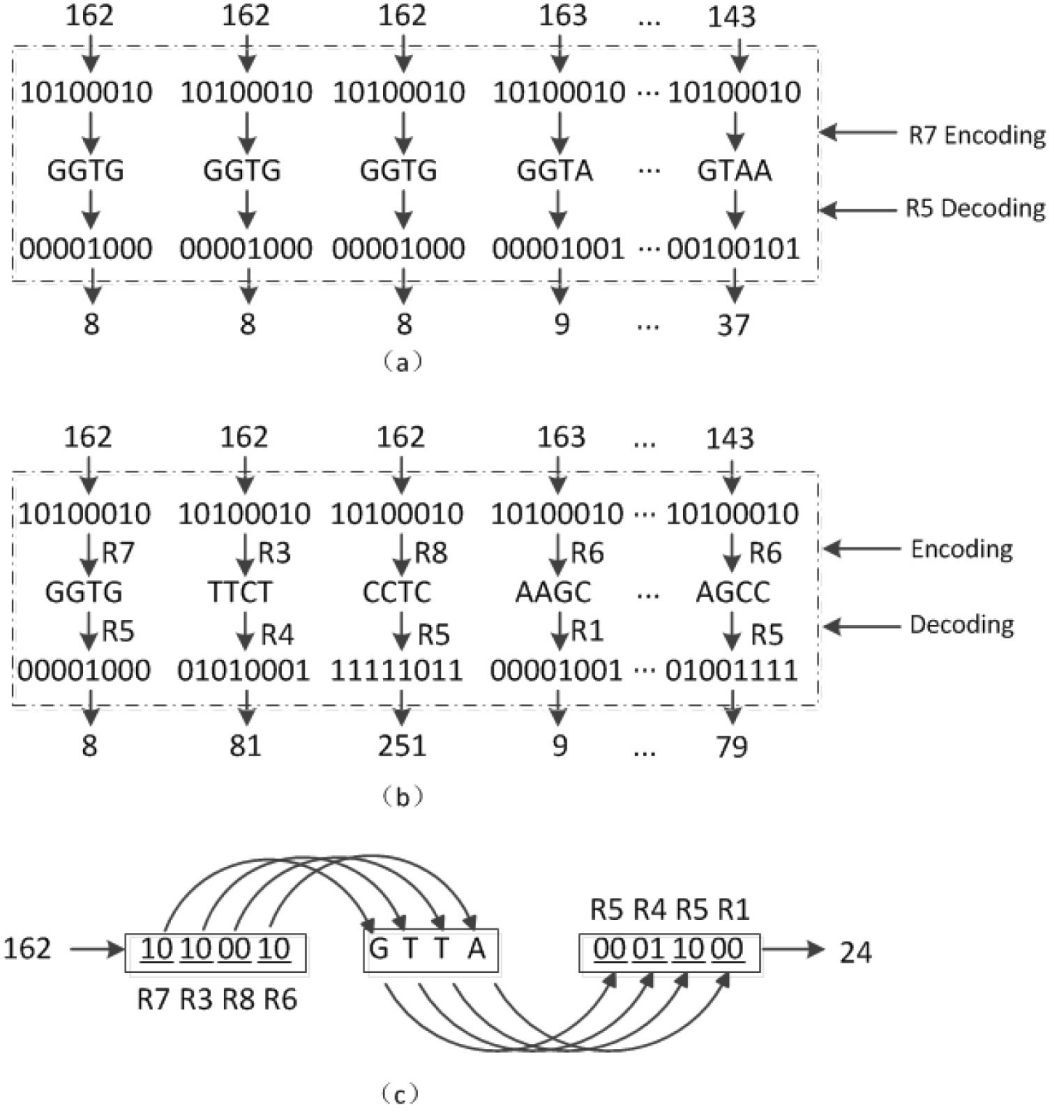
Decomposition diagram of dynamic coding (a) by row, (b) by pixel, and (c) by binary bit.

We assume that the chaotic sequences of the control encoding and decoding rules are E = {7,3,8,6…6} and D = {5,4,5,1…5}, respectively. Fig 4 presents a detailed description of the three dynamic encoding methods. Fig 4(a) depicts dynamic DNA encoding and decoding by row; because the first elements in E and D are 7 and 5, respectively, the first row of pixels for the “lena” image is encoded with R7 and decoded with R5. Similarly, the second row is encoded with R3 and decoded with R4. Fig 4(b) depicts dynamic DNA encoding and decoding by pixels. Each pixel in the first row of the “lena” image is encoded with R7, R3, R8, and R6…R6, and decoded with R5, R4, R5, and R1…R5. Fig 4(c) depicts dynamic DNA encoding and decoding by binary bit. Take the first pixel value (162) of the “lena” image as an example; the corresponding binary bit is “01010000.” In this case, R7, R3, R8, and R6 are used to encode every two binary bits, and R5, R4, R5, and R1 are used for decoding; finally, a pixel value of 24 was obtained.

**Fig 5:**
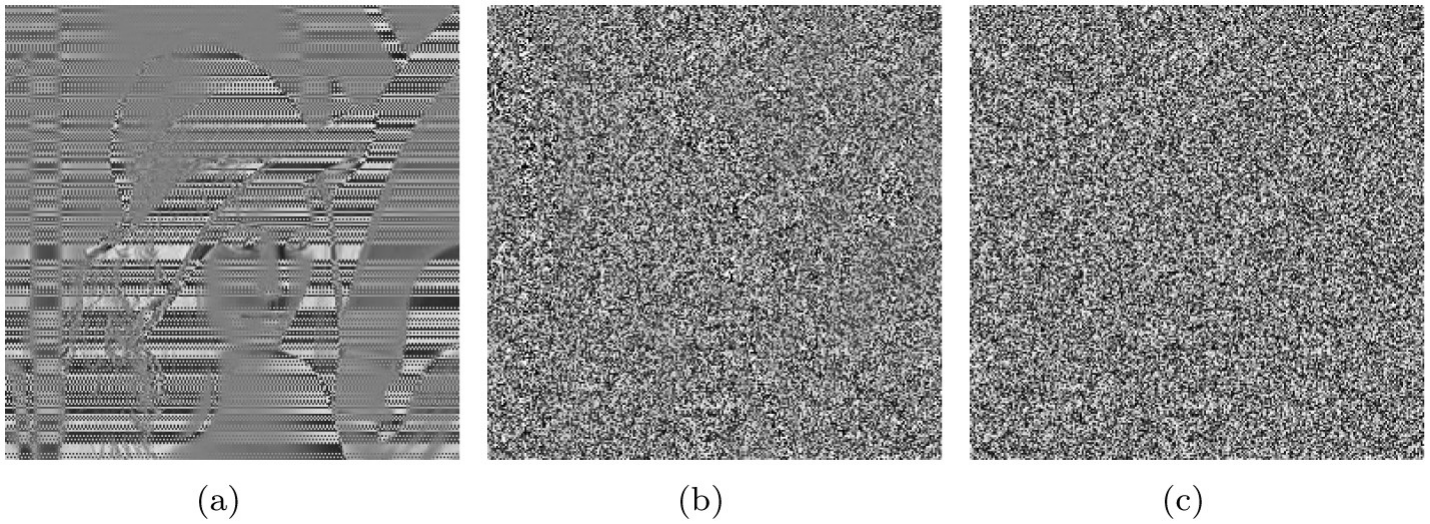
Three types of DNA dynamic encoding and decoding images for “lena”. Encrypted images using dynamic coding (a) by row, (b) by pixel, and (c) by binary bit.

Experiment 3: By using the logistic map described in Eq (3), the initial values x0 = 0.35, u1 = 3.95, y0 = 0.38, and u2 = 3.92 are chosen to generate two chaotic sequences of 1×256, and map them to the integer interval [1, 8] to obtain the encoding rule E and decoding rule D. Then, we implement the three dynamic DNA coding methods using E and D to encode and decode “lena” gray scale images, respectively. The encrypted images are shown in Fig 5(a)–5(c).

Experiment 4: Eqs (4), (6) and (8) are used to calculate the base distribution, information entropy, and histogram variance of Fig 5(a)–5(c) obtained in Experiment 3. Further, the time complexity of the algorithm and the maximum distance between the base distribution and 25% are calculated. The results of this experiment are presented in Table 5.

Fig 4 indicates that the correlation of the image pixel cannot be reduced by converting (162,162,162,…) to (8,8,8…) after the encoding and decoding operations using dynamic coding by row. However, dynamic coding by pixel and binary bit can reduce the correlation of the image pixels. In Fig 5, the contour of the original image is clearly seen in the image processed using dynamic encoding and decoding by row, but completely invisible in the images processed using dynamic encoding and decoding by pixel and binary bit. Table 5 shows that the base distributions of all these three methods are close to 25%. Moreover, the base distribution of dynamic coding by binary bits is much closer to 25% compared with that of the other two methods. The maximum distance between the base distribution and 25% is 0.04% for this method; additionally, the information entropy of its encrypted image is 7.9976, which is very close to eight. Furthermore, its histogram variance is the least, but the complexity order is highest.

**Table 5:** Comparative characteristics of different dynamic coding methods.

Comparative characteristic	Coding by row Dynamic coding	By pixel Dynamic coding	By binary bit
AP	24.50%	24.90%	24.98%
TP	24.65%	24.90%	25.04%
CP	25.42%	25.33%	24.97%
GP	25.43%	24.87%	25.01%
Information entropy	7.4895	7.8846	7.9976
Histogram variance	36177	11159	217.008
Complexity order	*O*(8*MN* + 2*M*)	*O*(10*MN*)	*O*(16*MN*)
The max distance of base distribution and 25%	0.50%	0.33%	0.04%

In summary, the implementation of dynamic coding by binary bit is more complex than that of the other two methods. Nevertheless, dynamic coding by binary bit performs better encryption, has higher degree of scrambling, and can resist exhaustive attacks.

### 3.3 Image encryption based on different types of base complement operations

The complement operation of a DNA sequence under chaotic sequence control is a common encryption tool for changing the pixel values of an image. According to the principle of base complementary in Table 2, two methods are available for base diffusion. The first method selects one of the six rules randomly, and then uses Eqs (2) and (17) to select the corresponding complementary bases [39, 43]; this method is called the static regular base complement method in this study. The other diffusion method [48] involves two steps: (a) selecting the complement rule for each base by chaotic sequence (i.e., to randomly select a rule from among R1 to R6 from Table 2), and (b) selecting the corresponding complement base using Eqs (2) and (17); this method is called the dynamic regular base complement method in this study. Moreover, [16] adopted a single base direct complement method (for details, see Section 2.2). Therefore, we classified the existing image encryption algorithms based on DNA complement into three: (1) single base direct complement method, (2) static regular base complement method, and (3) dynamic regular base complement method. The selection of the complement rule is expressed in the equation below:

$$\begin{array}{c} x_{i}=\{\begin{array}{cc} x_{i}, & if\;L(i)=0\\ D(x_{i}), & if\;L(i)=1\\ D(D\left(x_{i}\right)), & if\;L(i)=2\\ D(D\left(D\left(x_{i}\right)\right)), & if\;L(i)=3 \end{array} \end{array}$$ (17)

where L(i) is a chaotic sequence mapped to the integer region [0, 3], and *x*_*i*_ and *D*(*x*_*i*_) are the same as those in Eq (2). For the complement rule R1, if the base of the current position is *x*_*i*_ = *A*, and *ifL*(*i*) = 3, the complementary base of *x*_*i*_ is *D*(*D*(*D*(*x*_*i*_))). We can obtain the base as G (Table 2); in other words, the complement of A is G.

To comprehensively compare the above two categories involved in the three complementary methods, in the simulation of the above three methods, we use the logistic chaotic sequence generated by the same initial value to control the position of the complementary base. In addition, to avoid the interference of dynamic coding, we use R1 from Table 1 for encoding and decoding.

**Fig 6:**
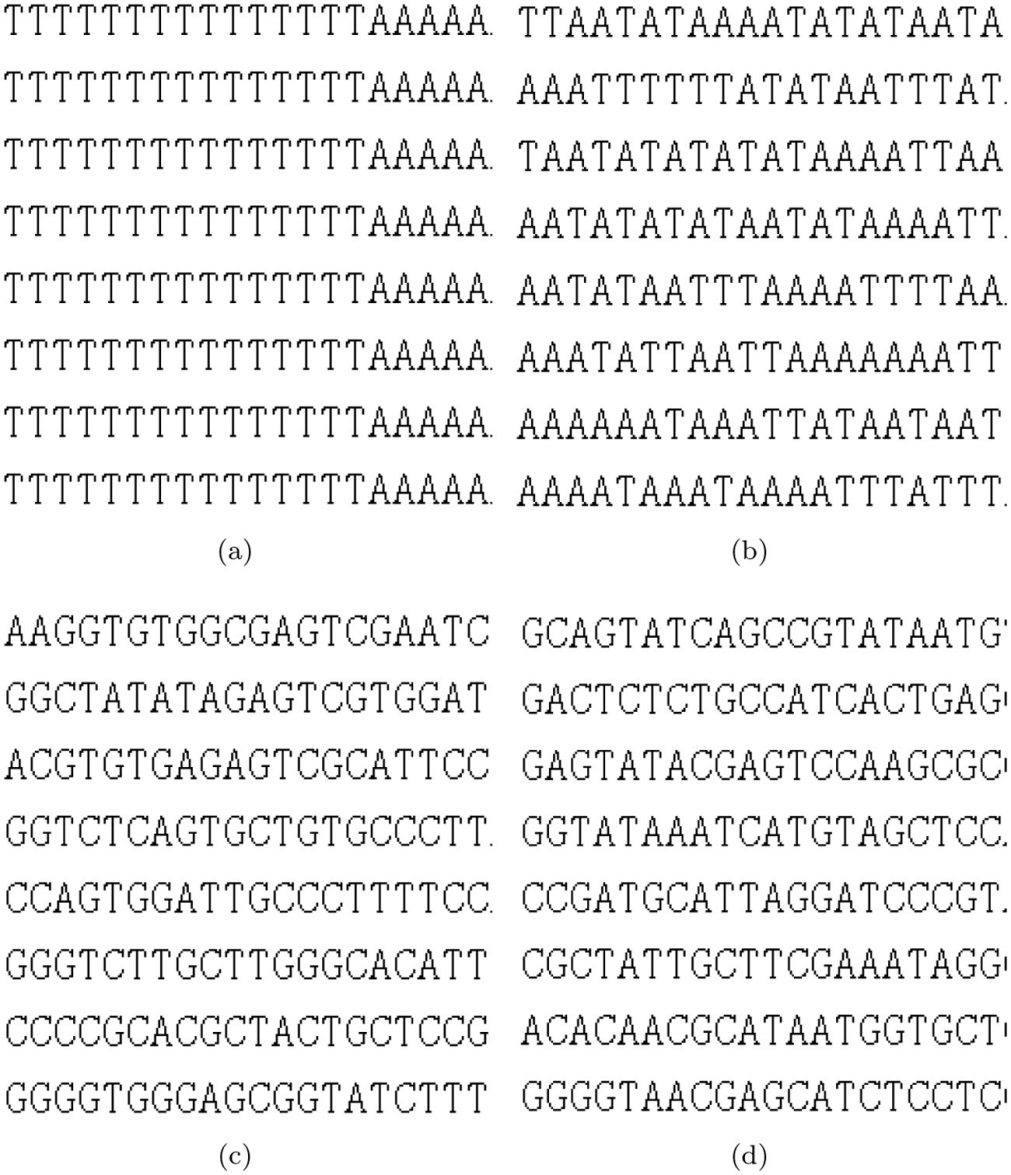
Variation of the partial bases in the first row of the“lena” image for the three complementary methods. (a) Variation of partial bases in the first row of the “lena” image, (b) variation of DBCO, (c) variation of SRCO, (d) variation of DRCO.

**Fig 7:**
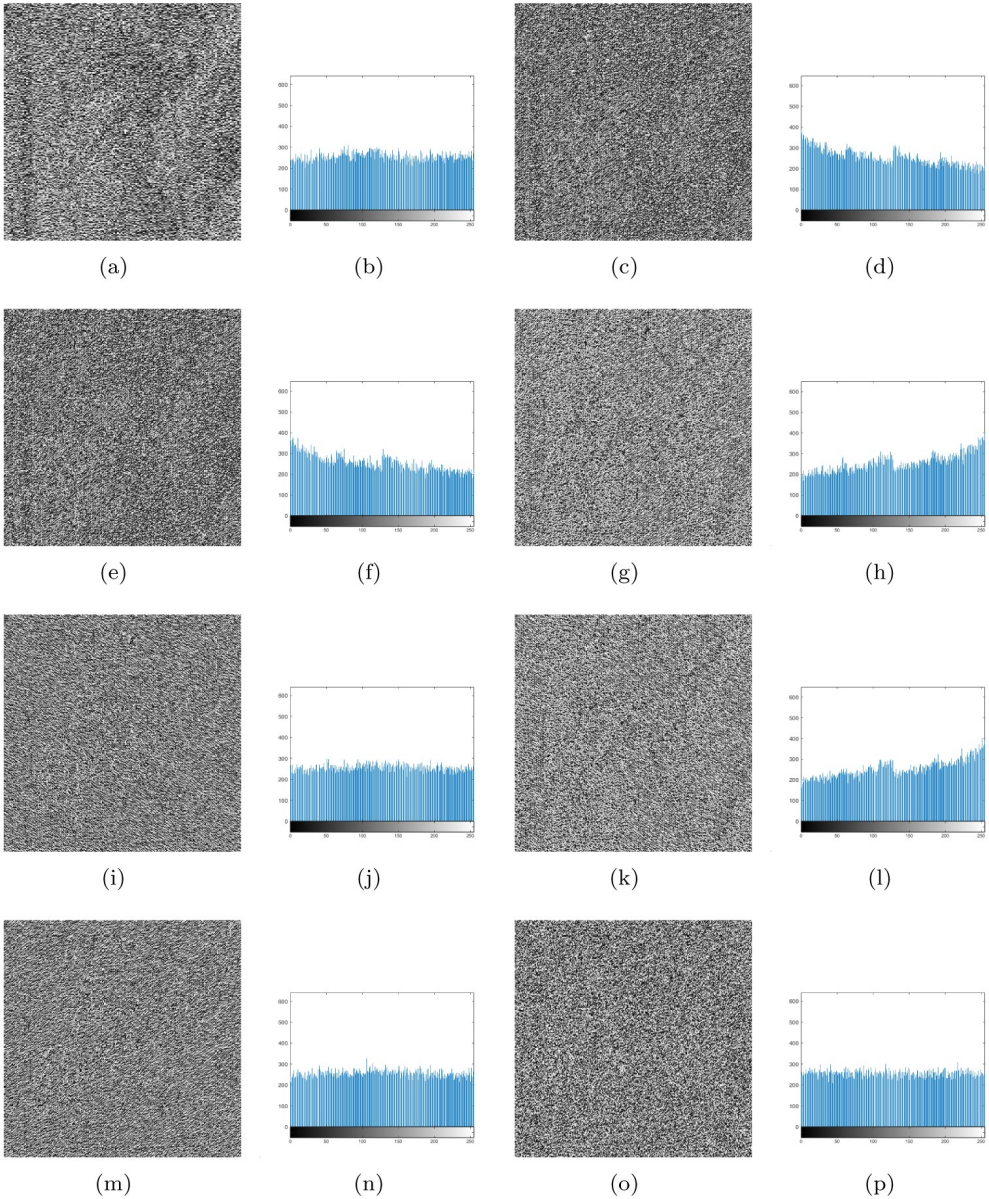
Encrypted images and histograms of “lena” for the three complementary methods. Encrypted images and histograms obtained by (a) and (b) DBCO, (c)—(n) SRCO (static rule is R1–R6 in Table 4), and (o)—(p) DRCO.

Experiment 5: The methods of Zhang [16] and Jian [44] were applied to the single base direct complement and the static regular base complement operation for the “lena” gray scale image of 256×256, using the logistic system with the initial values x0 = 0.38 and u1 = 3.95. Similarly, using the logistic system, the initial values x0 = 0.35, u1 = 3.95, y0 = 0.38, and u2 = 3.92 were taken to perform the dynamic regular base complement operation on the “lena” gray scale image of 256×256, based on the ideas from [48]. Fig 6 shows the “lena” partial base sequence and complemented base sequence obtained by the three methods. Fig 7 shows the encrypted images and the histograms obtained by the three methods. Table 6 compares the three methods in terms of information entropy, the hamming distance of the corresponding position base, and histogram variance. We use “DBCO,” “SRCO,” and “DRCO” to denote the single base direct complement, static regular base complement, and dynamic regular base complement methods, respectively, in Table 6, Figs 6 and 7.

**Table 6:** Comparison of the three complementary methods.

complementary methods	DBCO	SRCO						DRCO
		R1	R2	R3	R4	R5	R6	
Information entropy	7.9955	7.9852	7.9824	7.9794	7.9967	7.9803	7.9966	7.9969
Hamming distance	158812	204683	204683	204683	204683	204683	204683	204683
Histogram variance	405.2422	1619.6	1637.7	1927	296.0078	1840.7	312.1875	279.3750

From Fig 6, it is seen that the base diffusion of the corresponding position with static regular base complement operation or dynamic base complement operation is better than that of the direct base complement operation. Visual observation of Fig 7(a), 7(c), 7(e), 7(g), 7(i), 7(k), 7(m) and 7(o) shows that the encrypted images of the static regular base complement and the dynamic regular base complement methods are completely different from the original image, whereas the contour of the original image is seen in the image encrypted by the direct base complement method. Analyzing the histograms of Fig 7(b), 7(d), 7(f), 7(h), 7(j), 7(l), 7(n), and 7(p) shows that the histogram distributions for direct base complement and dynamic regular base complement are more uniform. The six histograms of the static regular base complement method showed three forms. The histograms of R1 and R2, R3 and R5, R4 and R6 have similar shapes, and the other four regular distributions are not uniform, except the histograms of R4 and R6. Here, R1–R6 were obtained from Table 2. A comparison of the information entropy and hamming distance of the three methods indicates that the information entropy of the dynamic regular base complement method is the largest, and the static regular base complement is not very stable. The hamming distance of the static regular base complement and dynamic regular base complement methods is the same and larger than that of the direct base complement method, because the chaotic sequence of the control complement operation is the same. The smaller the histogram variance, the better the encryption. Further, the variance value of the dynamic regular base complement method is the least of all methods.

In summary, the dynamic regular base complement operation performs better encryption, and has higher diffusion degree of pixels. Moreover, the complexity of this algorithm makes it robust to image encryption attacks.

### 3.4 Image encryption based on different DNA sequence algebra operations

Based on the discussion in Section 2.1, image pixels can be represented as DNA sequences by encoding. These DNA sequences can be changed by using different DNA sequence algebra operations. When the DNA sequence is changed, the pixel values of the image are disrupted. Thus, the addition, subtraction, and XOR algebra operations of the DNA sequences are widely used in image encryption. Table 3 shows that the rows and column bases corresponding to A and G of the addition and XOR operations are the same, and that the two operations are very similar. The main difference is that the addition operation is irreversible, whereas the XOR operation is reversible. Zhang et al. [12] used chaotic mapping and the DNA addition operations to perform image encryption. [18, 19] indicated that the encrypted image in [12] cannot be restored back to the original image. [59] also has the problem of irreversibility of the addition operation. To overcome this disadvantage, the DNA sequence matrix of the original grayscale image is usually added to that generated by the chaotic sequence [29, 30, 34, 60]. In case of a color image, the image is divided into three channel matrices (R, G, and B), and the channel matrix is transformed into DNA sequences, which are then added [14, 38]. The XOR operation also mainly follows the above two ideas. For example, the DNA sequence matrix of the original image and the DNA matrix which is generated by the chaotic system are carried out using the XOR operation [61–63]. Reference [64] implemented the XOR operation between the three channel DNA matrices in the color image. To compare the encryption performance of the addition and XOR operations, we do not scramble the original gray image. We use R1 in Table 1 to encode the original image, and then perform the addition and XOR operations using the DNA matrix produced by the chaotic sequence. The experimental procedure is as follows.

**Fig 8:**
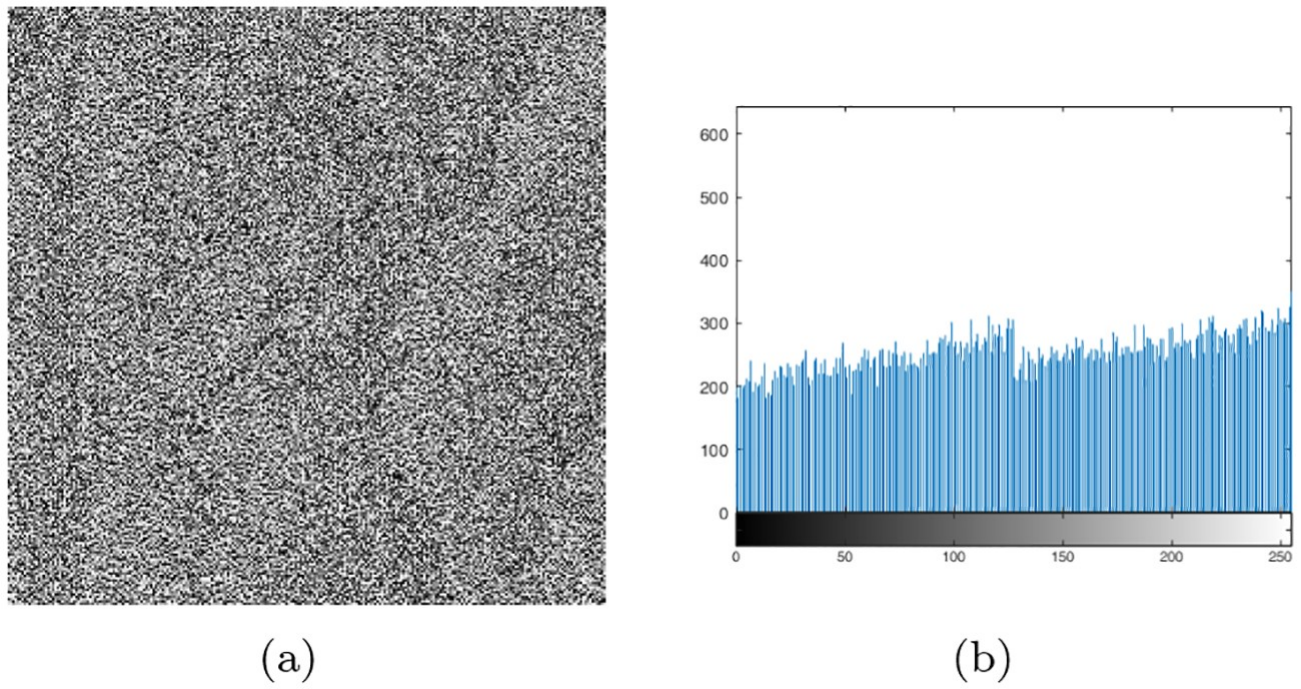
Encrypted image and histogram of addition operation. (a)Encrypted image of ‘lena’. (b) Histogram of encrypted image.

**Fig 9:**
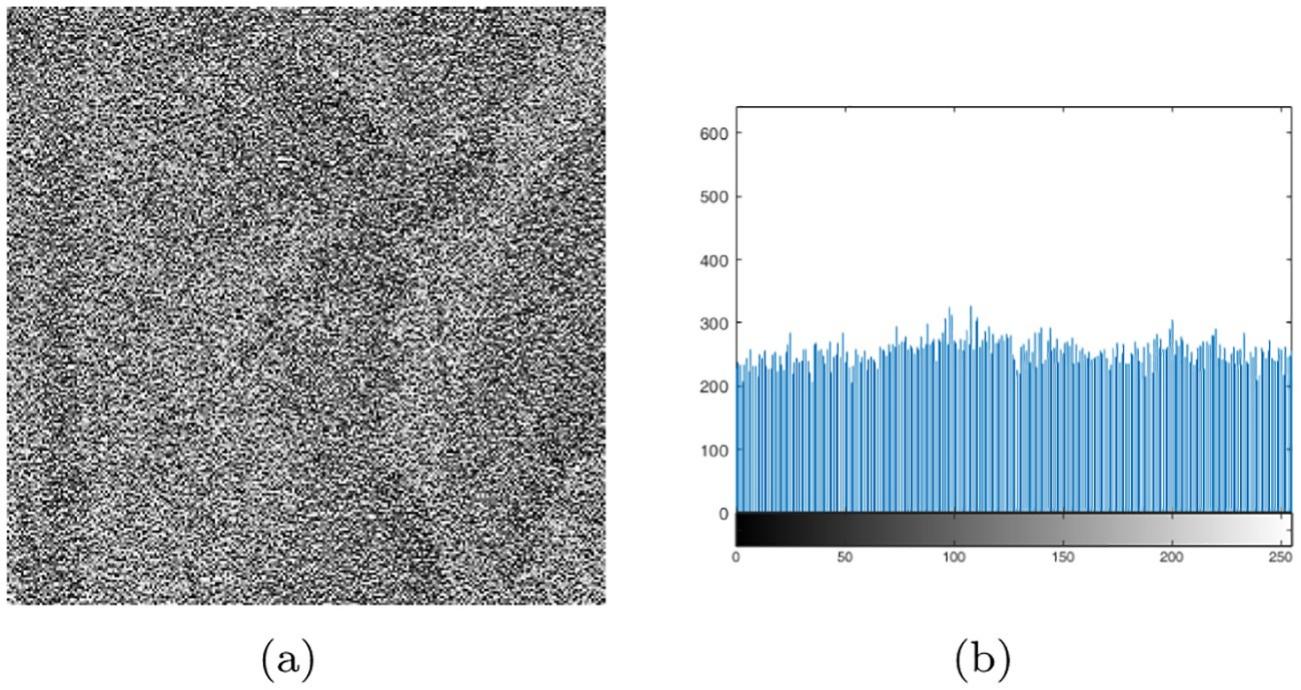
Encrypted image and histogram of XOR operation. (a) Encrypted image of “lena.” (b) Histogram of the encrypted image.

Experiment 6: The initial values x0 = 0.38 and u1 = 3.95 are chosen to generate the chaotic sequences of X_256×256×8_ using the logistic system. If x(i) > = 0.5, x(i) = 1, and if x(i) < 0.5, x(i) = 0; here, x(i) is an element of X. Then, the X sequence is encoded according to the fixed coding rule R1 in Table 1 to obtain the DNA matrix L, whose size is 256,256×4. The original “lena” image is also encoded into a DNA matrix P according to the fixed coding rule R1 in Table 1. We performed P+L and P XOR L operations by using the addition and XOR rules defined in Table 3. Finally, we obtained the encrypted image after decoding and reconstructing (Figs 8 and 9). To our knowledge, these methods have been adopted in [29, 30, 61–63].

In Figs 8(a) and 9(a), the contours of the original “lena” image can be seen, because the original image was not scrambled before the addition and XOR operations. A comparison of Figs 8(b) and 9(b) show that the histogram distribution of the encrypted images obtained by the XOR operation is more uniform. Table 7 indicates that the information entropy of the XOR operation is closer to eight. Because the two methods use the same chaotic mapping with the same parameters, they have the same hamming distance, and approximately 85% of their bases have changed (221592/(256×256×4)×100% = 85%).

**Table 7:** Comparison of addition and XOR operations.

DNA algebra operations	Original image	Addition operation	XOR operation
Information entropy	7.4895	7.9897	7.9953
Hamming distance	/	221592	221592
Fixed point ratio	/	0.14%	0.05%
histogram variance	40068	934.7500	424.3594

The smaller fixed point ratio and histogram variance indicate a better degree of disturbance. Clearly, the fixed point ratio and the histogram variance of the XOR operation are smaller as observed from Table 7. This indicates that the encryption of the DNA XOR operations is better than that of DNA addition.

### 3.5 Image encryption based on combinations of multiple DNA operations

Recently, combining DNA coding mechanisms and different DNA algebraic operations (such as addition and subtraction, and XOR and complement) to perform image encryption based on DNA coding has attracted the attention of scholars. Chai [65] used 2D logistic mapping to dynamically encode a plaintext image by pixels, and then used a cyclic displacement scrambling DNA matrix. Then, the addition and XOR operations were performed on the scrambled DNA matrix and the DNA matrix generated by the chaotic system under the new key updated by the hamming distance of the plaintext image. Finally, the encrypted image was obtained by decoding and recombining. Zhang [39] used Lorenz to generate chaotic sequences, and obtained a, b, and c after deforming. Then, Chen chaotic map was used to generate chaotic sequences, and A, B, C, and D were obtained after deformation. Further, the plain images were scrambled to obtain E1 using a and b, and E1 and C were dynamically encoded by binary bit using A and B, respectively, after that obtained E2 and E3. Addition operation was performed on E2 and E3 under c sequence, and E4 was obtained using D to perform the direct complement operation on E3 which is the result of addition, to finally obtain the encrypted image.

These methods perform good encryption, are highly secure, and can resist common attacks. However, most combination encryption methods are not secure, because most of them use the fixed coding rule, or the base is directly complemented, which was proven to be unsafe in Sections 3.1 and 3.3. Furthermore, some methods cannot restore the plaintext image, and cannot resist CPA and KPA. A detailed analysis is presented in Table 8.

**Table 8:** Analysis of image encryption based on combinations of multiple DNA operations.

Reference	Chaotic system	DNA Coding	Combination of DNA operations	Shortcomings
Zhang [12]	2D Logistic Logistic	Fixed coding	Addition Subtraction DBCO	The addition is irreversible
Liu [14]	Logistic	Fixed coding	Addition Subtraction DBCO XOR	Unable to resist the chosen plaintext attack, attacked by Belazi [20]
Zhang [17]	Lorenz Logistic	Fixed coding	XOR DBCO	Similar to the reference [14], the security is low and cannot resist the chosen plaintext attack
Wang [58]	CML Logistic	Fixed coding	Addition Subtraction SRCO	The security can be further improved
Jain [44]	Logistic 2D Logistic	Fixed coding	Addition Subtraction SRCO	The security can be further improved
Li [32]	Fractional-Order hyper chaotic system	Dynamic coding by binary bit	Addition Subtraction XOR	The algorithm has high complexity and cannotresist the chosen plaintext attack
Zhang [66]	Chen	Dynamic encoding by pixel decoding by fixed rule	Addition Subtraction XOR	Cannot resist the chosen plaintext attack, information entropy is lower
Suryadi [33]	Logistic	Fixed coding	Addition Subtraction DBCO XOR	Cannot resist the chosen plaintext attack, similar to the reference [14], the security is low
Zhang [34]	Logistic	Fixed coding	Addition Subtraction DBCO	Cannot resist the chosen plaintext attack
Dagadu [47]	Logistic Tent map	Dynamic coding by pixel	Addition Subtraction XOR	Cannot resist the chosen plaintext attack
Ahadpour [67]	Coupled chaotic maps lattices	Fixed coding	Addition Subtraction XOR DBCO	Similar to the reference [14], cannot resist the chosen plaintext attack

### 3.6 Further analysis and comparison of methods

The previous sections independently discussed the advantages and disadvantages of the methods. In this section, all the proposed methods are compared comprehensively. Image encryption based on DNA coding involves two main aspects: DNA coding mechanisms and DNA coding operation. We compare these two aspects individually.

#### 3.6.1 Comparison of DNA coding mechanisms

DNA coding (encoding and decoding) is the interface between image pixels and DNA sequences. Different DNA coding mechanisms yield different encryption performances. Therefore, choosing a better DNA coding method is a key step in the DNA image encryption process. The existing DNA coding methods are compared in Tables 4 and 5. The performance of the three dynamic DNA coding methods is better than that of fixed DNA coding. Among all the dynamic DNA coding methods, the dynamic coding by binary bit has the best performance; the information entropy is 7.9976, which is very close to 8. Further, its histogram variance is the least, suggesting that the histogram distribution of the encrypted image is the most uniform for this method than for the other methods. It has the least maximum distance between the base distribution rate and 25%, indicating that the base distribution is the most uniform for this method than for the other methods. However, due to calculation by binary bit, this algorithm is more complex than the others. We believe that the current speed of the computer is totally affordable for this method.

#### 3.6.2 Comparison of DNA coding operations

The existing DNA encoding operations include individual DNA operations and the combination of multiple operations. We compared them based on information entropy, histogram variance, correlation coefficient, and time complexity (Tables 9 and 10). Table 9 indicates that the information entropy of DRCO is much closer to 8 than that of the other DNA coding operations. Further, its histogram variance and correlation coefficient (very close to 0) are the least. However, it has the highest complexity order. The order of superiority of the other DNA coding operations is as follows: SRCO (Rule 4 and Rule 6 from Table 6) >DBCO>XOR>addition. This order can be used to determine if a specific DNA operation is reasonable. Because DRCO exhibits the best performance of all the base complement methods, the combination of DRCO with other DNA operations attracts our attention. All DNA operations in this section used the same experimental data and environment as those in the previous sections. Table 10 shows that DRCO + Addition is better than Addition + DRCO, DRCO + XOR is better than XOR + DRCO, and XOR + Addition is better than Addition + XOR. Table 9 shows that DRCO>Addition, DRCO>XOR, and XOR>Addition.

**Table 9:** Comparison of the performance of DNA coding operations.

DNA coding operation		Information entropy	Histogram variance	Correlation coefficient			Complexity order
				Horizontal	Vertical	Diagonal	
Base complement	DBCO	7.9955	405.2422	0.4117	-0.1642	-0.0685	*O*(16*MN*)
	SRCO	7.9967(max)	296.008(max)	-0.0412	-0.3457	-0.0435	*O*(16*MN*)
	DRCO	7.9969	279.3750	-0.0317	0.0010	0.0629	*O*(32*MN*)
Algebra operations	Addition	7.9897	934.7500	-0.0383	-0.1260	-0.0153	*O*(24*MN*)
	XOR	7.9953	424.3594	0.0238	-0.1646	-0.0018	*O*(24*MN*)

**Table 10:** Comparing the performance of combinatorial DNA operations.

Combination of DNA operation	Information entropy	Histogram variance	Correlation coefficient			Complexity order
			Horizontal	Vertical	Diagonal	
Addtion+DRCO	7.9967	297.1640	0.0158	-0.0159	-0.0147	*O*(48*MN*)
DRCO+Addtion	7.9969	279.0156	0.0156	-0.0094	0.0195	*O*(48*MN*)
XOR + DRCO	7.9970	273.2500	0.0086	-0.0051	0.0025	*O*(48*MN*)
DRCO+ XOR	7.9974	233.2500	-0.0033	0.0012	0.0022	*O*(48*MN*)
Addition+XOR	7.9969	281.2813	-0.0069	0.0248	0.0042	*O*(40*MN*)
XOR +Addition	7.9971	260.1094	-0.0081	0.0051	0.0019	*O*(40*MN*)
DRCO+XOR+Addition	7.9975	225.3203	-0.0031	0.0007	-0.0011	*O*(64*MN*)

These results show that when performing combinatorial DNA operations, better encryption performance can be obtained by placing the better operation before other operations. Table 10 indicates that DRCO + XOR is the best encryption method of all combinatorial operations, because DRCO and XOR operations occupy superior positions in the order DRCO>XOR>addition. If time complexity permits, the superposition of the operation with better performance will result in better encryption; for instance, DRCO + XOR + Addition>DRCO + XOR. This would indicate the direction for implementing DNA combinatorial operation encryption later.

### 3.7 Proposed DNA coding encryption scheme

Section 3.6.1 revealed that the dynamic DNA coding by binary bit is the best DNA coding method; Section 3.6.2 revealed that DRCO + XOR + Addition is the best DNA coding operation. Now, we attempt to develop a new encryption scheme by combining the optimal DNA coding and optimal DNA operation defined above. First, the original image is encoded by binary bit dynamically; then, DRCO + XOR + Addition operation is performed on the coding matrix. Finally, the encrypted image is obtained by decoding the DNA coding matrix to the binary sequence dynamically. The encryption process is the same as the decomposition process mentioned above, and is thus not discussed again. However, as described by Eqs (18) and (19), the key is treated as follows to make the proposed scheme more secure.

$$\begin{array}{c} a_{0}=\frac{\sum_{i=1}^{M}\sum_{j=1}^{N}A\left(i,j\right)}{10\times M\times N} \end{array}$$ (18)

$$\begin{array}{c} x_{terminal}=a_{0}+x_{initial}\left(k\right) \end{array}$$ (19)

**Fig 10:**
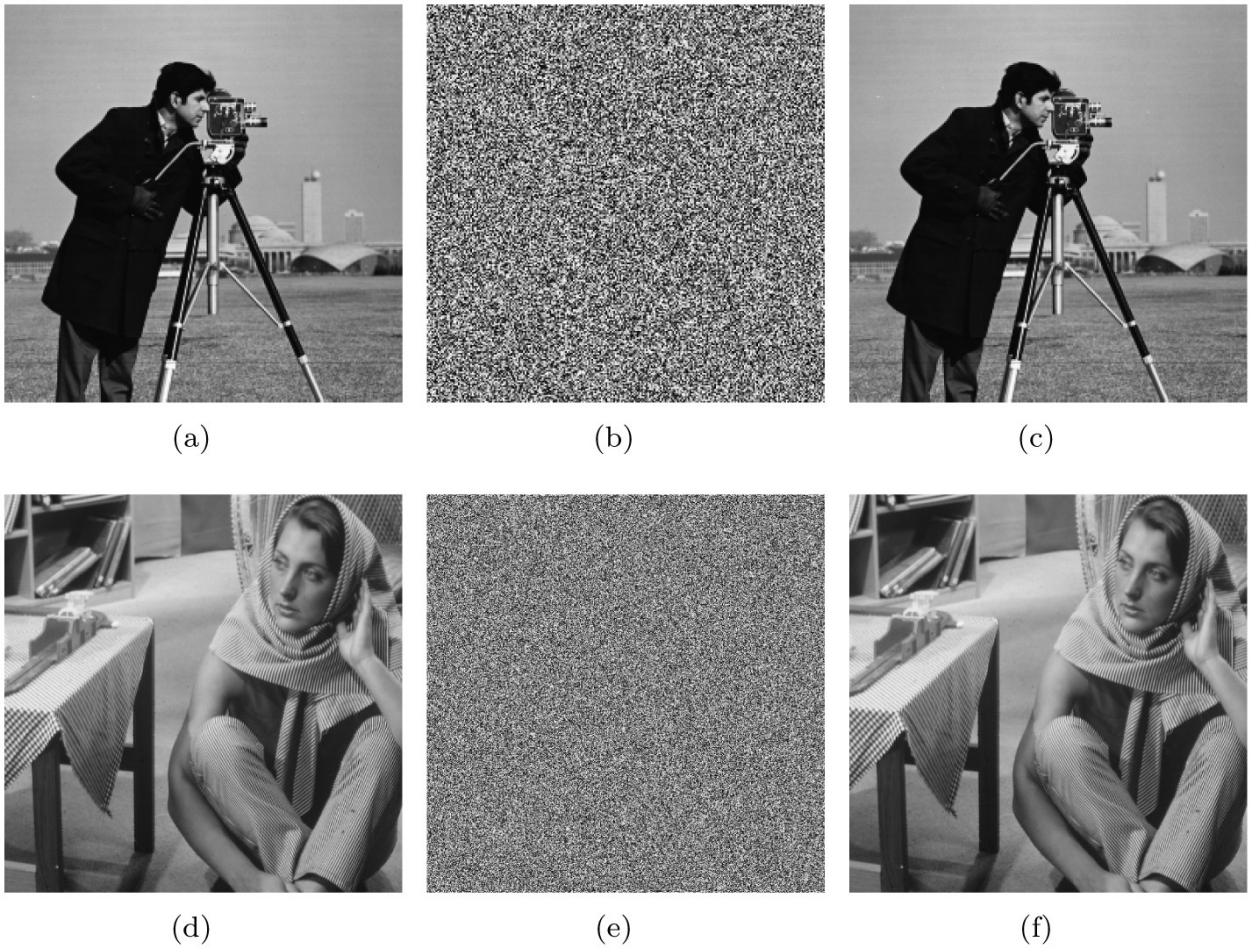
Encrypted image and decrypted image produced by the proposed scheme. (a) Original image of “Cameraman,” (b) Encrypted image of “Cameraman,” (c) Decrypted image of (b); (d) Original image of “Woman,” (e) Encrypted image of “Woman,” and (f)Decrypted image of (e).

We apply the proposed algorithm to ten standard gray-scale images, of which seven are 256×256 and three are 512×512. The encrypted images of “Cameraman” and “Woman” are shown in Fig 10. Apparently, the encrypted images are difficult to be recognized, but only from a visual perspective. Next, we evaluate the proposed algorithm in terms of information entropy, resistance to exhaustive attacks, resistance to statistical attacks, and resistance to differential attacks, and conduct a NIST randomness test.

#### 3.7.1 Analysis of global and local information entropy

To analyze the information entropy more accurately, we calculate both the global information entropy and local information entropy of different scales. This is because the local information entropy can effectively overcome the weaknesses of the global information entropy, such as inaccuracy, inconsistency, and low efficiency. Table 11 shows that the global information entropy and local information entropy of each block are 16×16 and 32×32, respectively (here, k = 30 and L = 256). Further, all global information entropy are very close to 8, and the average local entropy of 16×16 and 32×32 is 7.1766 and 7.8033, respectively; these values are close to 7.1749 and 7.8087, which are the local information entropy of the random images in [68]. Table 12 compares the developed encryption scheme with the existing encryption schemes. The comparison indicates that the information entropy obtained by our scheme is higher, and the encrypted image is similar to the ideal random image.

**Table 11:** Global and local information entropy of the encrypted images.

Image	size	Global entropy	Local entropy 16×16	Local entropy 32×32
lena	256×256	7.9979	7.1790	7.8064
Airplane	256×256	7.9974	7.1767	7.8028
Cameraman	256×256	7.9973	7.1867	7.8014
Sailboat	256×256	7.9974	7.1730	7.8021
Parrot	256×256	7.9973	7.1750	7.8024
Monarch	256×256	7.9973	7.1702	7.8049
starfish	256×256	7.9971	7.1765	7.8034
couple	512×512	7.9994	7.1754	7.8046
woman	512×512	7.9993	7.1790	7.8029
Man	512×512	7.9993	7.1742	7.8022

**Table 12:** Comparison of information entropy of the proposed and the existing methods.

Scheme	size	Entropy	Scheme	size	Entropy
Proposed	256×256	7.9974	Proposed	512×512	7.9993
Ref [69]	256×256	7.9970	Ref [70]	512×512	7.9588
Ref [71]-Ahmad’s	256×256	7.9801	Ref [69]	512×512	7.9990
Ref [71]-Niu’s	256×256	7.9888	Ref [72]	512×512	7.9985
Ref [71]	256×256	7.9972	Ref [73]	512×512	7.9993
Ref [74]	256×256	7.99615	Ref [75]	512×512	7.9986

#### 3.7.2 Key sensitivity analysis (resistance to exhaustive attacks)

The key is determined to be sensitive or insensitive depending on whether the encryption scheme can resist exhaustive attacks. We use NPCR and UACI to analyze the difference between two encrypted images with a key (x0) difference of 10^−14^. Table 13 lists the NPCR and UACI values of the encrypted images before and after minor changes to the key. NPCR = 99% and UACI = 33% are the benchmark values [53]. Table 13 reveals that all the NPCR and UACI values of our method are larger than the benchmark values. Table 14 compares our algorithm with the existing algorithms, and reveals that the NPCR and UACI values of our algorithm are larger than those of the existing methods. Therefore, our algorithm has high key sensitivity and is robust against exhaustive attacks.

**Table 13:** Results of key sensitivity analysis.

image	NPCR(%)	UACI(%)
lena	99.61	33.60
Cameraman	99.60	33.60
Parrot	99.60	33.59
woman	99.60	33.48
Man	99.59	33.45
Average	99.60	33.54

**Table 14:** Comparison of key sensitivities of the proposed and the existing methods.

Scheme	NPCR(%)	UACI(%)
Proposed	99.60	33.54
Ref [76](average)	99.60	33.46
Ref [77]	99.64	33.31
Ref [71]	99.57	33.49
Ref [78]	99.58	33.37

#### 3.7.3 Resistance to statistical attacks

**Fig 11:**
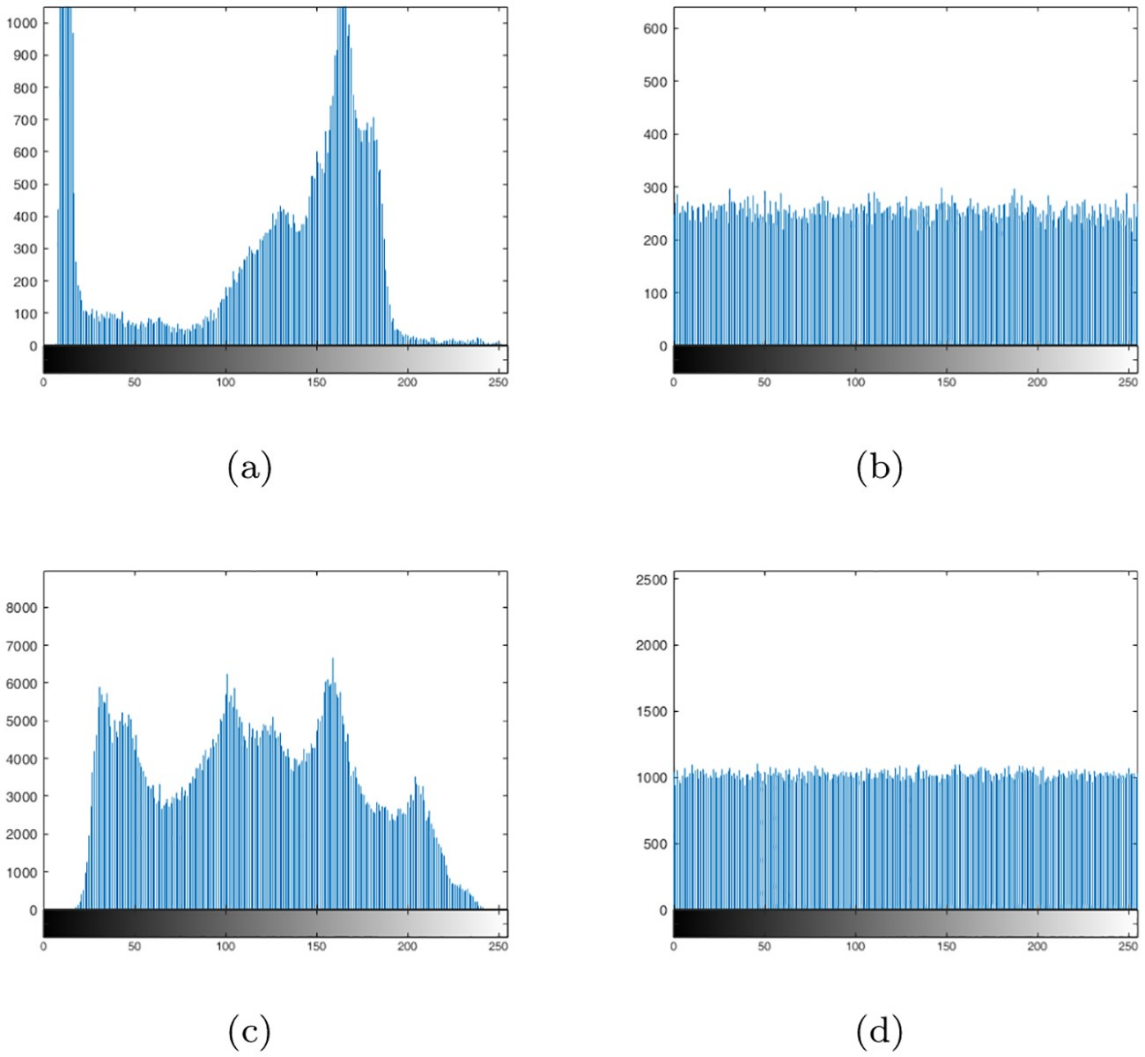
Histograms of the original and the encrypted images. (a)Histograms of “Cameraman,” (b) Histograms of encrypted image of “Cameraman,” (c))Histograms of “Woman,” (d) Histograms of encrypted image of “Woman.”

Here, we discuss the effectiveness of the proposed algorithm in resisting statistical attacks. Cryptoanalysts generally use the distribution of histograms and the correlation between adjacent pixels to count important information in order to find information related to plaintext images [79, 80]. Therefore, the histograms of “Cameraman” and “Woman” are shown in Fig 11, and the histogram variances of all 10 standard original and encrypted images are listed in Table 15. Further, we use Eqs (10)–(13) to calculate the correlation coefficients of 10 standard gray-scale images and the corresponding encrypted images (Table 16).

**Table 15:** Histogram variances of the original and encrypted images.

Image	size	Original	encrypted
lena	256×256	40068	192.3438
Airplane	256×256	177300	236.6328
Cameraman	256×256	110970	244.6875
Sailboat	256×256	68597.5	232.1094
Parrot	256×256	26464.5	241.9922
Monarch	256×256	40207	246.9297
starfish	256×256	16292	260.6642
couple	512×512	1387200	881.4033
woman	512×512	382200	1015.1
Man	512×512	1201300	1011.4

**Table 16:** Correlation coefficients of the original and encrypted images.

Image	size	Original image			Encrypted image		
		H	V	D	H	V	D
Lena	256 × 256	0.9421	0.9718	0.9160	0.0010	−6.8640 × 10^−5^	-0.0014
Airplane	256 × 256	0.8337	0.8950	0.9029	0.0014	0.0011	−7.0240 × 10^−4^
Cameraman	256 × 256	0.9306	0.9609	0.9080	1.0600 × 10^−4^	-0.0021	0.0019
Sailboat	256 × 256	0.8652	0.9162	0.8156	0.0016	-0.0013	7.8222 × 10^−4^
Parrot	256 × 256	0.9581	0.9389	0.9100	0.0013	0.0011	8.7092 × 10^−4^
Monarch	256 × 256	0.9354	0.9265	0.9060	0.0029	8.9210 × 10^−4^	−5.6692 × 10^−5^
starfish	256 × 256	0.9598	0.9611	0.9360	5.6808 × 10^−4^	-0.0026	−1.4678 × 10^−4^
couple	512 × 512	0.9456	0.9554	0.9115	−1.5336 × 10^−4^	−8.0640 × 10^−4^	0.0015
woman	512 × 512	0.8969	0.9576	0.8827	−4.3208 × 10^−4^	0.0017	−1.4784 × 10^−4^
Man	512 × 512	0.9632	0.9684	0.9385	0.0031	-0.0015	9.4602 × 10^−4^

Apparently, the distributions of the histograms for encrypted images are very uniform, and the histogram variance of all encrypted images is much lower than that of the original image. The correlation coefficients in the horizontal, vertical, and diagonal directions of the encrypted images are very close to 0, suggesting that the correlation between adjacent pixels is very low. Therefore, the proposed scheme can resist statistical attacks very well.

#### 3.7.4 Resistance to differential attacks

To attack an encryption algorithm, a cryptographic attacker searches for loopholes by detecting the sensitivity of the keys to plaintext. If the keys are more sensitive to plaintext, it is difficult to perform a differential attack. Similar to that in Ref. [80, 81], we use NPCR and UACI to calculate the difference value after encrypting two plaintext images with very little differences. Table 17 lists the NPCR and UACI values for five standard grayscale images, and indicates that the averages of the NPCR and UACI values are higher than the corresponding benchmark values. Further, we compare our scheme with other existing methods in Table 18; Table 18 shows that our scheme is highly superior to the existing methods.

**Table 17:** NPCR and UACI values of two plaintext images with very little differences (1st pixel value+1).

image	NPCR(%)	UACI(%)
lena	99.62	33.44
Cameraman	99.61	33.43
Parrot	99.62	33.48
woman	99.60	33.50
Man	99.61	33.57
Average	99.61	33.48

**Table 18:** Comparison of NPCR and UACI values of the proposed and the existing methods.

Scheme	NPCR(%)	UACI(%)
Proposed	99.61	33.48
Ref [82]	99.59	33.45
Ref [71]	99.59	32.46
Ref [74]	99.60	30.15
Ref [70]	99.52	33.51
Ref [83]	99.61	33.46

#### 3.7.5 NIST randomness test

The SP800-22 test package of the National Institute of Standards and Technology (NIST) can detect the randomness of digital information, and is currently the most authoritative method for detecting the randomness of binary sequences [81, 84, 85]. Each value in this package is tested successfully only if it is greater than 0.01. Further, to preserve generality, we detect the randomness of both encrypted grayscale images and color images of three channels, and the results are presented in Table 19. Table 19 shows that all test values are greater than 0.01, and that the results are “success.” This shows that our algorithm can obtain random sequences by encrypting grayscale and color images.

**Table 19:** NIST randomness test of encrypted images.

Test	P values for different color layer of ciphered image				Results
	Gray	Red	Green	Blue	
Frequency	0.050840	0.093618	0.106726	0.343429	success
Block frequency	0.982411	0.935871	0.896638	0.739019	success
Rank	0.645533	0.032864	0.232596	0.109194	success
Run(M = 10000)	0.434548	0.025371	0.075586	0.475306	success
Long runs of ones	0.217490	0.492130	0.629059	0.577289	success
Linear complexity	0.334230	0.082060	0.776813	0.059248	success
Overlapping templates	0.234659	0.909920	0.490812	0.753541	success
Non overlapping templates	all P value>0.01	all P value >0.01	all P value >0.01	all P value >0.01	success
FFT	0.418761	0.463844	0.659148	0.423150	success
Approximate entropy	0.033674	0.786329	0.901310	0.279076	success
Universal	0.855914	0.563772	0.912692	0.232085	success
Serial P values 1	0.044550	0.163760	0.458336	0.533797	success
Serial P values 2	0.088164	0.367355	0.233775	0.686673	success
Cumulative sums forward	0.085808	0.183481	0.178746	0.442739	success
Cumulative sums reverse	0.024093	0.176673	0.088104	0.403578	success

## 4 Shortcomings of existing methods and suggested improvements

### 4.1 Shortcomings

Section 3 indicates that the existing methods have some shortcomings. First, the existing DNA coding mechanisms are fragile. Although DNA coding is a crucial step in encryption, most of the existing coding methods use fixed coding. Section 3.1 proved that fixed coding yields poor encryption performance, has poor resistance to exhaustive attacks, and has non-uniform distribution of bases. Although some studies have used dynamic coding, most of them are row-by-row (image block) or pixel-by-pixel dynamic coding. These two methods are not as secure as dynamic coding by binary bit. Second, improper application of the DNA sequence addition operation, resulting in irreversibility of the image encryption method. The inverse operation of DNA sequence addition is the DNA sequence subtraction operation. Therefore, if addition is performed between the pixels of an original image, the DNA subtraction operation cannot be performed, and it would be difficult to decrypt an encrypted image. Third, the security of the DNA complement operations is poor. In recent years, various studies have widely used the method of DNA complement to diffuse the pixel values. However, most of them used the direct base complement or static regular base complement method. Section 3.3 showed that the static regular base complement method comprises three different encryption forms. Except for the fourth and the sixth rule, the information entropy of the other static regular complement methods is low, and the direct base complement method exhibits poor encryption performance. Fourth, because the combinatorial DNA operations are selected arbitrarily, image encryption based on combinations of multiple DNA operations is not highly secure. Fifth, the diffusion capacity of DNA bases is poor. Most studies only use the relevant theory of DNA coding to encrypt images and ignore the diffusion of bases, making their methods vulnerable to CPA and KPA. Sixth, the existing methods use chaotic systems combined with DNA coding to achieve image encryption, whose security (i.e., key space, key sensitivity, and degree of image scrambling) thus depends on the security of the chaotic system. Furthermore, parallelism and high storage of DNA computing were not applied to these image encryption methods.

### 4.2 Future direction for improvements

We propose the following improvements to alleviate the above shortcomings. The first suggestion is related to using the DNA dynamic coding mechanism to transform an original image into a DNA sequence matrix, for which dynamic coding by binary bit can be preferred. Table 5 shows that the information entropy of dynamic coding by binary bit reaches 7.9976, and its base distribution is very uniform, which are beneficial as good factors to the successfully begin the encryption process. Second, the DNA addition operation can be replaced with DNA XOR operation to perform image encryption. Table 3 indicates that the DNA XOR operation is very similar to the addition operation. Its main advantage is that it is reversible, and makes the algorithm simple. By comparing the relevant data, we can solve the problem of irreversible addition, and also obtain good encryption performance. Third, selecting the dynamic regular base complement method to improve the diffusion capacity of pixels is preferable. Fourth, choose a reasonable and effective DNA combination operation to change the pixel values of images based on Table 10. Fifth, use the information related to plaintext as a part of encryption key, such as combining the hamming distance of the DNA sequence from the plaintext image with the encryption key (chaotic initial value) to form the final key, or combining DNA dynamic coding with a chaotic system. This can improve the diffusion capacity of the DNA base, and effectively resist the CPA and KPA. Sixth, in addition to using more secure chaotic systems combined with DNA coding to perform image encryption, researchers should combine the DNA coding methods effectively to improve the security of the DNA coding encryption methods.

Future studies should emphasize the use of DNA computing parallelism and large storage in image encryption to quickly encrypt many images and even video files, while ensuring security.

## 5 Conclusion

This study first reviewed the existing DNA coding-based image encryption methods. Image encryption based on DNA coding was classified into five types, depending on the type of DNA coding: DNA fixed coding, DNA dynamic coding, different types of base complement operation, different DNA sequence algebraic operations, and combinations of multiple DNA operations. All these methods and other existing methods were compared and explained. Furthermore, we combined the optimal coding mechanism with the optimal DNA coding operation to develop a new encryption scheme, and demonstrated its effectiveness and security. Finally, the shortcomings of the existing image encryption methods and the future direction for improvement were discussed. In the future, we will study the advantages and disadvantages of image encryption methods based on different combinations of DNA coding and dynamic DNA operations. We will also study the influence of different chaotic systems on DNA coding schemes.

## Supporting information

S1 File (ZIP)
