# Expression of Concern: New insights into the existing image encryption algorithms based on DNA coding

**DOI:** 10.1371/journal.pone.0353144

**Published:** 2026-07-07

**Authors:** The PLOS One Editors

The *PLOS One* Editors issue this Expression of Concern for this article [1] because of concerns identified about the peer review process. Readers are advised to interpret the article [1] with caution.

The Editors have no evidence of any author involvement in the peer review concerns.

Additionally, Reference 70 in [1] is retracted.
