# Supplementary figures and images for: New insights into the existing image encryption algorithms based on DNA coding
Source: PLoS One. 2020 Oct 23;15(10):e0241184. doi: 10.1371/journal.pone.0241184 (PMC7584250; doi:10.1371/journal.pone.0241184)

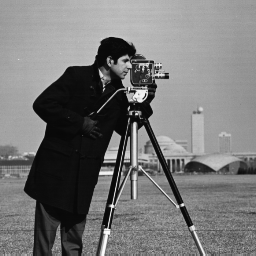

Supplement: S1 File — (ZIP) [file pone.0241184.s001.zip › supporting imformation/Fig10(a).tif]

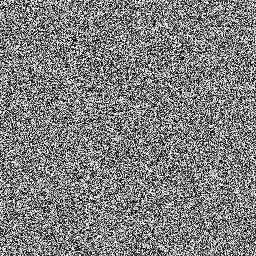

Supplement: S1 File — (ZIP) [file pone.0241184.s001.zip › supporting imformation/Fig10(b).tif]

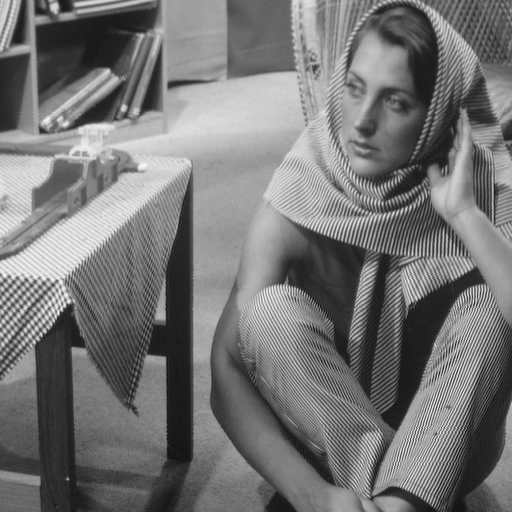

Supplement: S1 File — (ZIP) [file pone.0241184.s001.zip › supporting imformation/Fig10(d).tif]

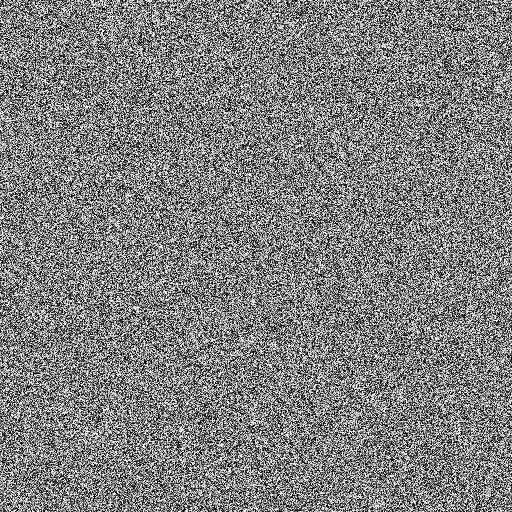

Supplement: S1 File — (ZIP) [file pone.0241184.s001.zip › supporting imformation/Fig10(e).tif]

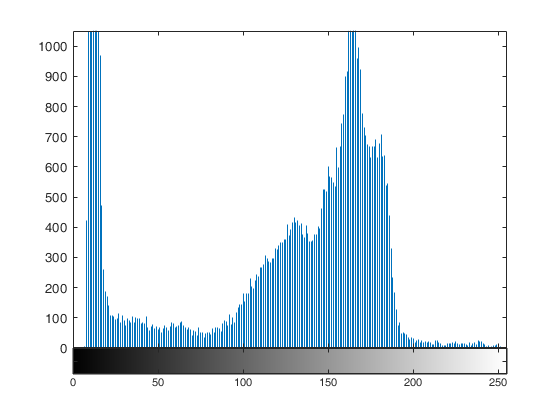

Supplement: S1 File — (ZIP) [file pone.0241184.s001.zip › supporting imformation/Fig11(a).tif]

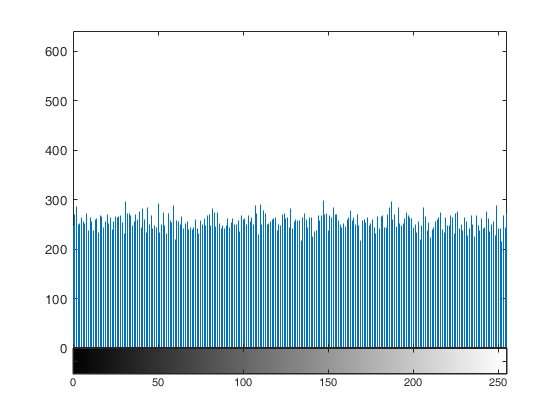

Supplement: S1 File — (ZIP) [file pone.0241184.s001.zip › supporting imformation/Fig11(b).tif]

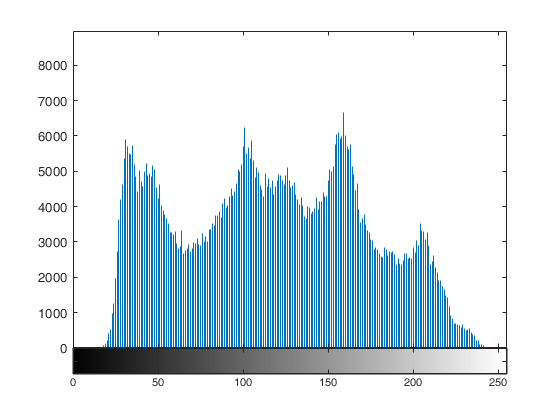

Supplement: S1 File — (ZIP) [file pone.0241184.s001.zip › supporting imformation/Fig11(c).tif]

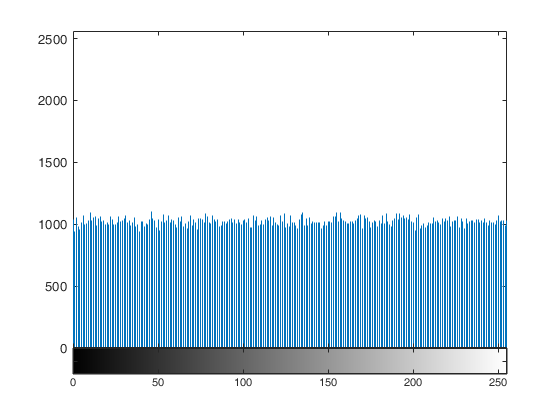

Supplement: S1 File — (ZIP) [file pone.0241184.s001.zip › supporting imformation/Fig11(d).tif]

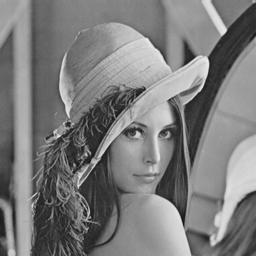

Supplement: S1 File — (ZIP) [file pone.0241184.s001.zip › supporting imformation/Fig3(a).tif]

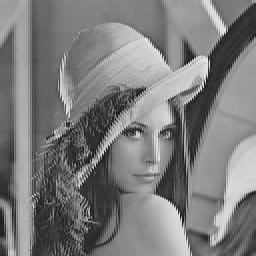

Supplement: S1 File — (ZIP) [file pone.0241184.s001.zip › supporting imformation/Fig3(b).tif]

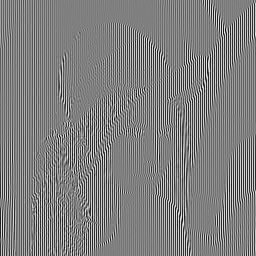

Supplement: S1 File — (ZIP) [file pone.0241184.s001.zip › supporting imformation/Fig3(c).tif]

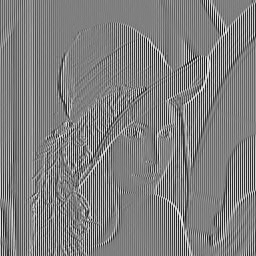

Supplement: S1 File — (ZIP) [file pone.0241184.s001.zip › supporting imformation/Fig3(d).tif]

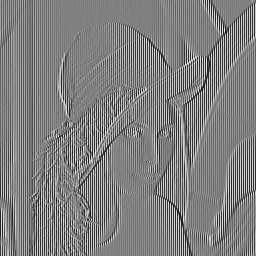

Supplement: S1 File — (ZIP) [file pone.0241184.s001.zip › supporting imformation/Fig3(e).tif]

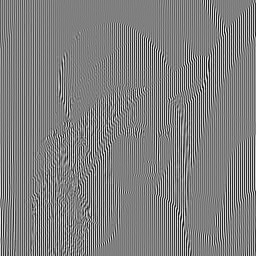

Supplement: S1 File — (ZIP) [file pone.0241184.s001.zip › supporting imformation/Fig3(f).tif]

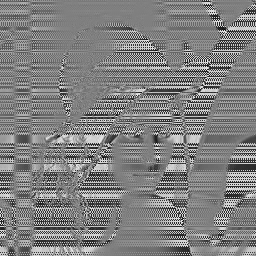

Supplement: S1 File — (ZIP) [file pone.0241184.s001.zip › supporting imformation/Fig5(a).tif]

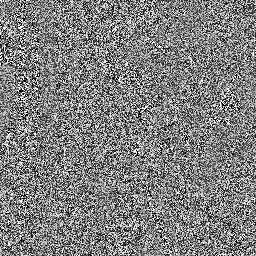

Supplement: S1 File — (ZIP) [file pone.0241184.s001.zip › supporting imformation/Fig5(b).tif]

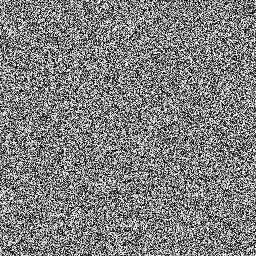

Supplement: S1 File — (ZIP) [file pone.0241184.s001.zip › supporting imformation/Fig5(c).tif]

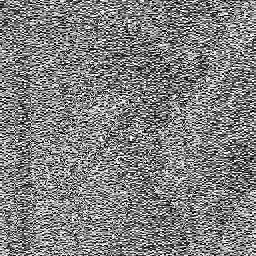

Supplement: S1 File — (ZIP) [file pone.0241184.s001.zip › supporting imformation/Fig7(a).tif]

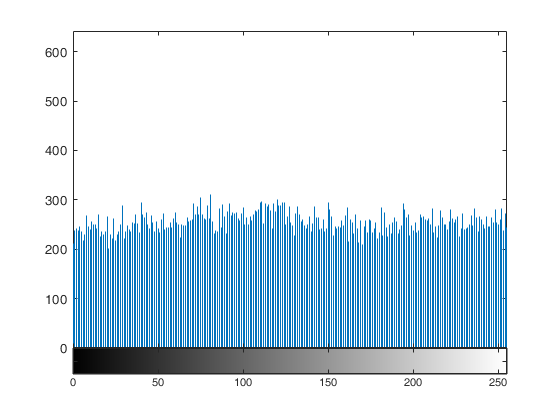

Supplement: S1 File — (ZIP) [file pone.0241184.s001.zip › supporting imformation/Fig7(b).tif]

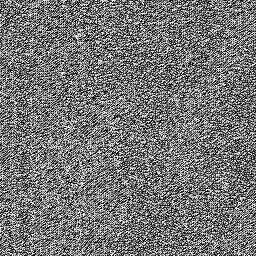

Supplement: S1 File — (ZIP) [file pone.0241184.s001.zip › supporting imformation/Fig7(c).tif]

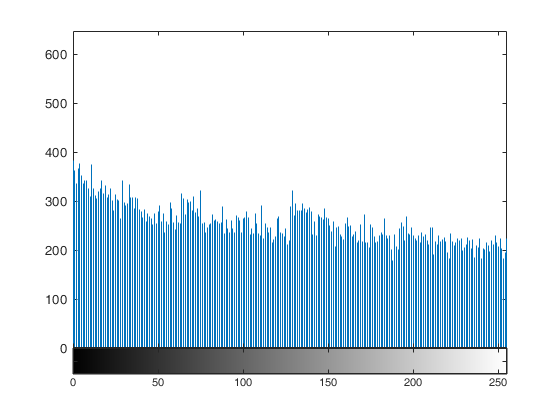

Supplement: S1 File — (ZIP) [file pone.0241184.s001.zip › supporting imformation/Fig7(d).tif]

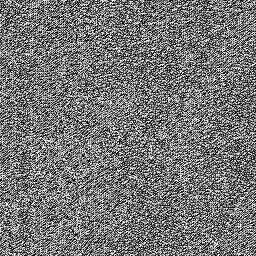

Supplement: S1 File — (ZIP) [file pone.0241184.s001.zip › supporting imformation/Fig7(e).tif]

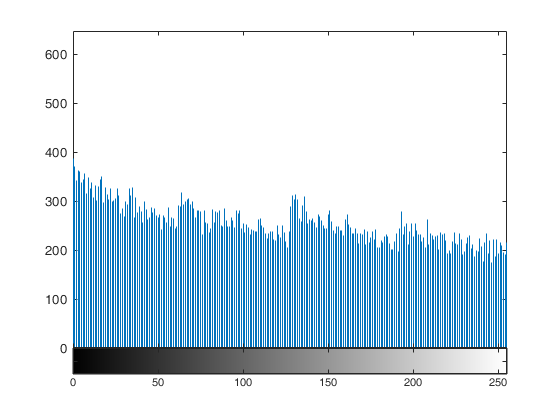

Supplement: S1 File — (ZIP) [file pone.0241184.s001.zip › supporting imformation/Fig7(f).tif]

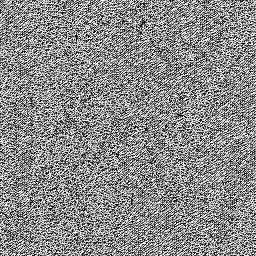

Supplement: S1 File — (ZIP) [file pone.0241184.s001.zip › supporting imformation/Fig7(g).tif]

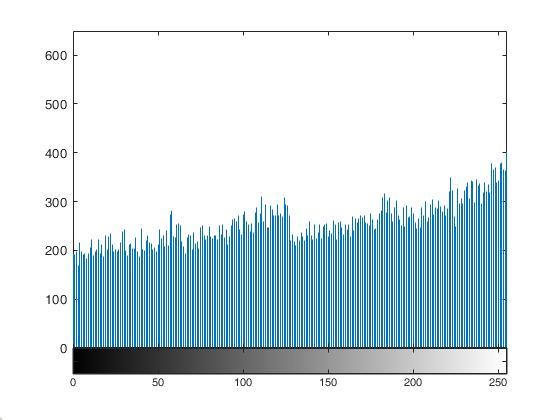

Supplement: S1 File — (ZIP) [file pone.0241184.s001.zip › supporting imformation/Fig7(h).tif]

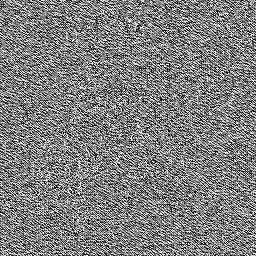

Supplement: S1 File — (ZIP) [file pone.0241184.s001.zip › supporting imformation/Fig7(i).tif]

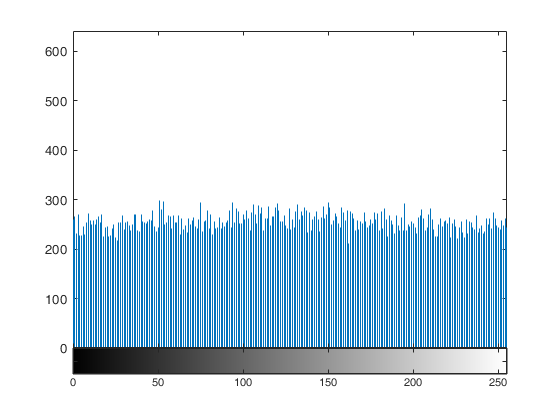

Supplement: S1 File — (ZIP) [file pone.0241184.s001.zip › supporting imformation/Fig7(j).tif]

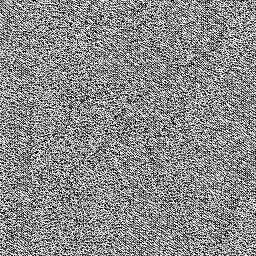

Supplement: S1 File — (ZIP) [file pone.0241184.s001.zip › supporting imformation/Fig7(k).tif]

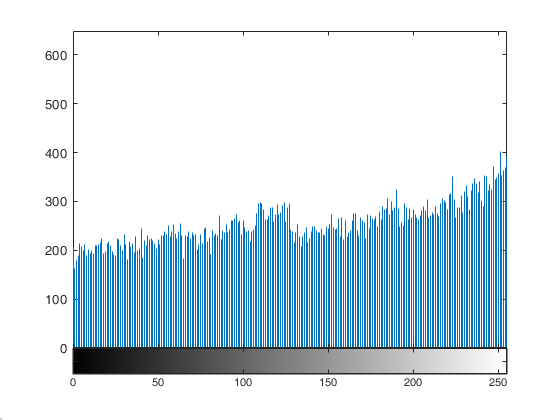

Supplement: S1 File — (ZIP) [file pone.0241184.s001.zip › supporting imformation/Fig7(l).tif]

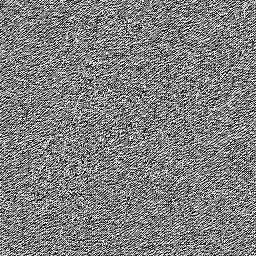

Supplement: S1 File — (ZIP) [file pone.0241184.s001.zip › supporting imformation/Fig7(m).tif]

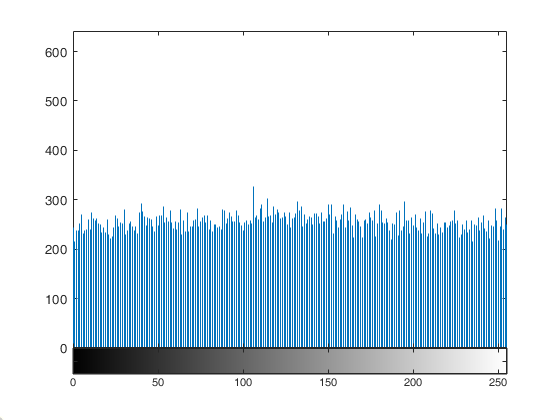

Supplement: S1 File — (ZIP) [file pone.0241184.s001.zip › supporting imformation/Fig7(n).tif]

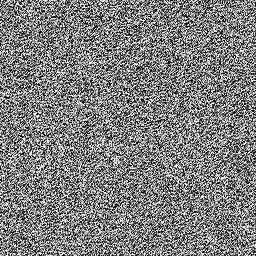

Supplement: S1 File — (ZIP) [file pone.0241184.s001.zip › supporting imformation/Fig7(o).tif]

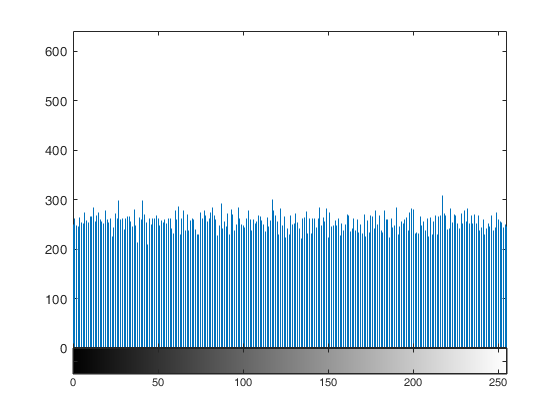

Supplement: S1 File — (ZIP) [file pone.0241184.s001.zip › supporting imformation/Fig7(p).tif]

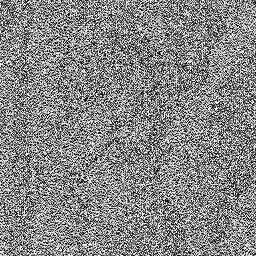

Supplement: S1 File — (ZIP) [file pone.0241184.s001.zip › supporting imformation/Fig8(a).tif]

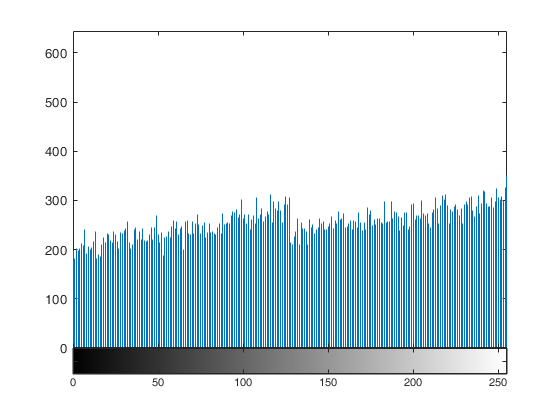

Supplement: S1 File — (ZIP) [file pone.0241184.s001.zip › supporting imformation/Fig8(b).tif]

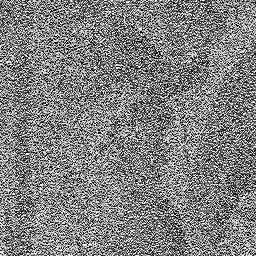

Supplement: S1 File — (ZIP) [file pone.0241184.s001.zip › supporting imformation/Fig9(a).tif]

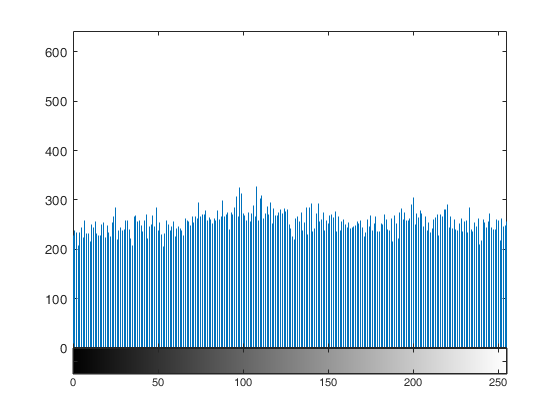

Supplement: S1 File — (ZIP) [file pone.0241184.s001.zip › supporting imformation/Fig9(b).tif]

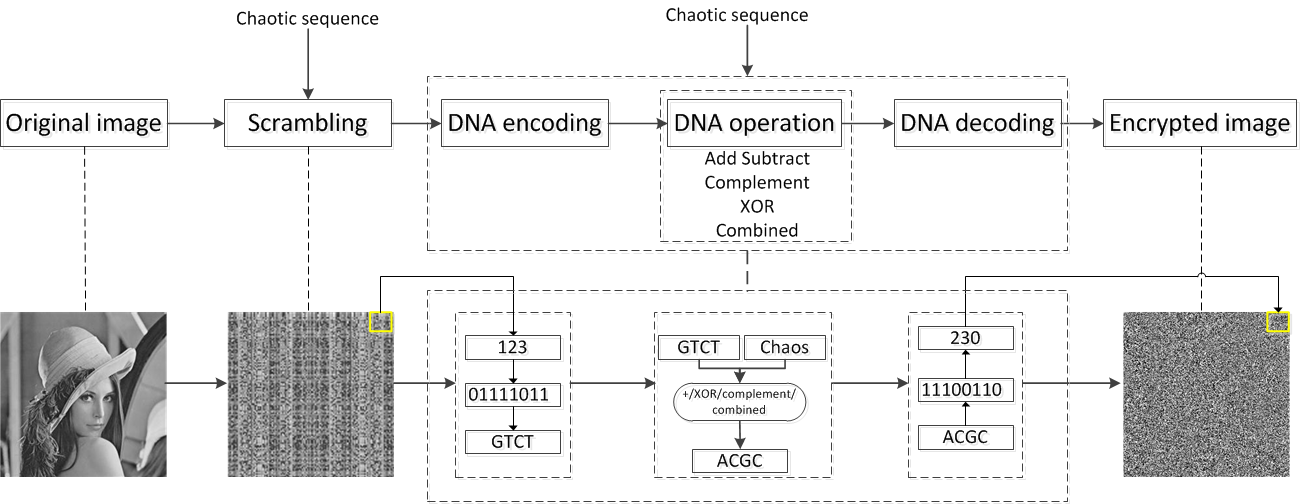

Supplement: S1 File — (ZIP) [file pone.0241184.s001.zip › supporting imformation/S1_Fig.tif]

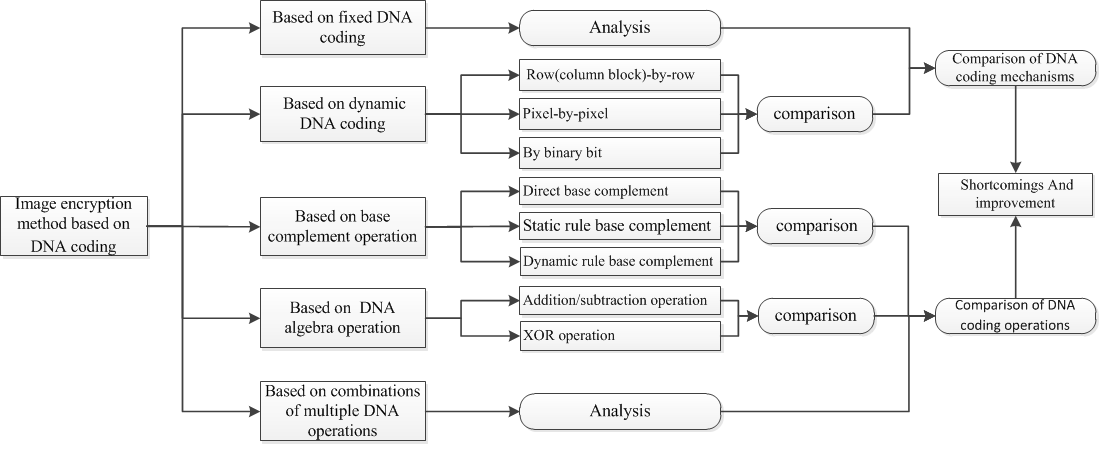

Supplement: S1 File — (ZIP) [file pone.0241184.s001.zip › supporting imformation/S2_Fig.tif]

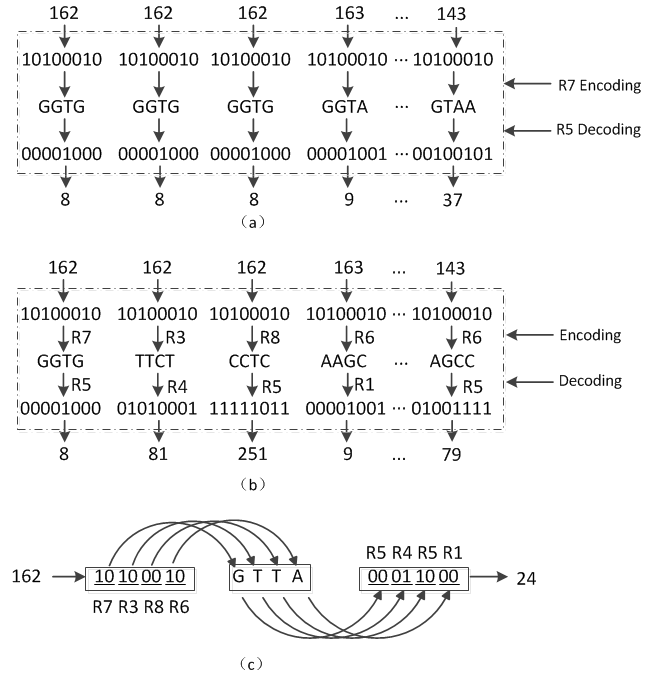

Supplement: S1 File — (ZIP) [file pone.0241184.s001.zip › supporting imformation/S4_Fig.tif]
